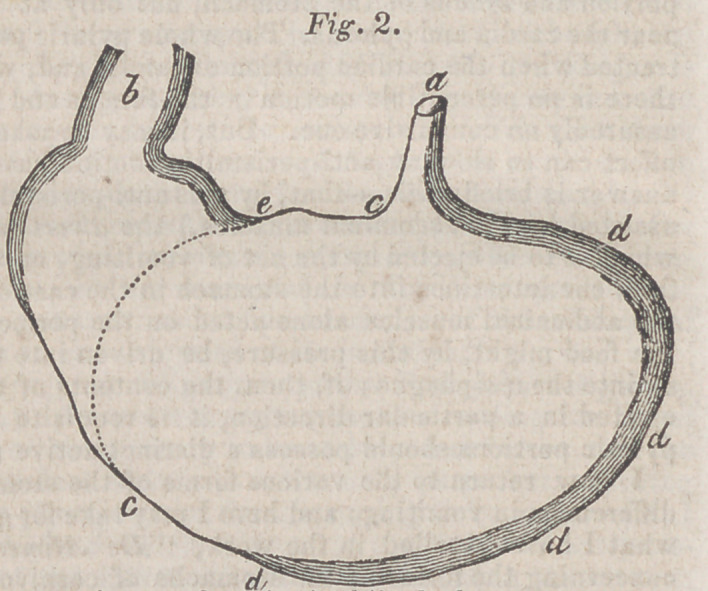# Analectic

**Published:** 1837

**Authors:** 


MISCELLANEOUS INTELLIGENCE.

### ANALECTIC, ANALYTICAL, AND ORIGINAL.

### I.—ANALECTIC.

*Ad Sylvas Nuncius.*

1. *On the Use of Belladonna as a Topical Application in Retention of Urine, Spasmodic Contractions of the Uterus, and in Strangulated Hernia.*—[The well known relaxant effects of belladonna on the iris, etc., has naturally led to its use in cases where spasm was known or assumed to exist in other parts. M. Guerin, of Bourdeaux, was the first, we believe, who employed it in spasmodic strictures of the urethra, in the form of ointment spread on a bougie; and he states that he found the same remedy, applied in the same manner, effectual in the case of strangulated hernia. Since then, belladonna has been frequently used topically in similar and analogous cases; and we shall here extract the heads of a few of the more recent which have met our eye in some of the foreign journals.]

*Efficacy of Extract of Belladonna in Retention of Urine.*—By M. Gerard, late chief Surgeon of the Hospital at Avignon.—Case 1. A lady, æt. 36, was delivered of her first child, after a long and severe labor, at one A. M., on the 16th November, 1834. Nine hours thereafter it was discovered that the urinary bladder was immensely distended and painful, no water having been passed since the commencement of the labor, and there being still an incapacity to do so. No attempt seems to have been then made to introduce the catheter; the surgeon contenting himself with ordering "vegetable lemonade, and an emollient poultice to the hypogastrium." At nine P. M., no urine having been passed, (now three days,) matters were of course worse, and then the surgeon seems for the first time to have thought of the catheter, but he could not succeed in its introduction, owing to what he terms "a manifest contraction of the urethra." Being deterred by the patient's debility from the use of general and local bleedings or the warm bath, M. Gerard prescribed an ointment composed of two drachms of Extract. Bellad. to one ounce of lard, and ordered it to be rubbed on the hypogastrium and labia. The first friction was made at midnight, the next at three A. M.; and shortly after this last the patient began to make water in small quantities, with much pain. The frictions were continued through the day, and the urine at length flowed plentifully.

Case. 2. A man, æt. 49, was attacked with retention of urine, accompanied with fever, for which he was copiously bled and leeched on two successive days, without relief. On the third day, frictions with the belladonna ointment were used on the hypogastrium and perineum. After the third friction there was a slight discharge of urine; and on the following day, the frictions being continued, the patient was completely relieved.

Case 3. A man, æt. 24, suffered very acute pains in the region of the bladder, attended with retention of urine for three days, the consequence of a severe blow. After the failure of general and local bleedings, and the warm bath continued for four hours, the belladonna ointment was had recourse to, and the urine flowed after the third application.

Case 4. A man, affected with stricture of the urethra for six years, called in M. Gerard in consequence of a suppression of urine, which had lasted four days, notwithstanding the employment of general and local bleeding, bathing, anodynes, etc. The belladonna ointment was ordered: after the first friction a slight flow of urine took place, and the relief was complete after the continuance of the friction for thirty-six hours.—*Journ. des Connaissances Med.Chir.*, *Mai*, 1835.

2. *Employment of Belladonna in Spasmodic Contractions of the Uterus, Urethra, and Inguinal Ring.* By M. Carre, chief Surgeon of the Military Hospital of Briancon.—Case 1. A lady was in labor of her third child, the waters had broken, and, as no progress was gained, the midwife attempted to dilate the os uteri by her fingers. This proceeding increased the irritation and contraction, and produced general convulsions. M. Carre, being called in, bled the patient and used the warm bath, but to no purpose. He then ordered the os tincæ to be rubbed with belladonna ointment every half-hour; and, after the third friction, the uterus became sufficiently dilated to permit the operation of turning, and the child was delivered, and lived. The ointment was made by rubbing up eight grammes of Ext. Belladonnæ with sixty-four grammes of cerate, and of this from two to four grammes were used each time.

Case 2. A woman, æt. twenty-one, was prematurely taken in labor at the eighth month. The waters had broken for some time, and, when M. C. was called, he found the os uteri so strongly contracted upon an arm of the fœtus, that he could not introduce his hand. Having first had recourse to bleeding, etc., the same ointment was applied, and, after the fourth friction, the dilatation was sufficient to permit the operation of turning, and the extraction of a dead child.

Case 3. A man had suffered from retention of urine for twenty-four hours, without any relief from bleeding and baths. The catheter could not be introduced beyond two inches, on account of the spasmodic contraction of the urethra. As the patient had been able to make water freely previously to the attack, M. C., believing the case merely spasmodic, prescribed the belladonna, which he applied by friction with the ointment on the glans, and by applying to the perineum a poultice made with decoction of the leaves, and further moistened with a solution of the extract. In an hour or two the urine began to flow slowly, and he was completely relieved in three hours.

Case 4. A man suffered a protrusion of voluminous inguinal hernia in attempting to lift a load. After ineffectual attempts at reduction, the use of bleeding, baths, etc., M. C. had recourse to M. Guerin's practice, introducing into the urethra a bougie covered with equal parts of cerate and extract of belladonna, and in half an hour's time he was able to reduce the hernia.

Case 5. A soldier, subject for some years to a hernia, and for which he used a truss, had the misfortune to break this in leaping a ditch, and his hernia protruded and became strangulated. He had been ill twenty-four hours, suffering great pain, vomiting, etc., when M. C. saw him. Bleeding, baths, and the taxis were tried in vain. The tumor was then rubbed with the belladonna ointment, and a cataplasm applied. The pains ceased in from half an hour to three quarters, and the taxis being then admissible, it proved readily successful.

Case 6. Another soldier suffered in the same way, and was relieved by the belladonna ointment after the failure of other means.—*Ib.*

3. *Two Cases of incarcerated Hernia cured by the use of Belladonna Ointment.* By Pietro Porta, M. D., of San Zenone.—Case I. A stout healthy man, æt. 50, upon lifting a heavy weight, was seized with a sudden pain, attended with tumor in the right iliac region. A medical man having recognized a crural hernia, bled the patient, and prescribed warm fomentations. The next day Signor Porta was called in, when the intense pain, meteorism, hiccough, vomiting, and obstipation, unrelieved by a second bleeding and the taxis, determined him to resort to the use of the belladonna, in the form of dried leaves ʒj. to lard ʒvj. This however could not be procured for a whole day, during which delay all the symptoms became much aggravated; nevertheless a few frictions with the ointment over the tumor caused it to disappear with all its attendant symptoms.

Case 2. This was supposed to be a case of omental inguinal hernia, and occurred in a child of five years old. The tumor was inelastic, doughy, and irregular, giving rise to no prominent symptoms of suffering, but still, after several days, remaining irreducible by the taxis and warm baths. The belladonna ointment, applied every two hours for three days, succeeded in effecting the reduction, after the failure of every other means.

[Although the majority of the foregoing cases are far from presenting positive evidence of the efficacy of belladonna as a relaxer of the spasm present or presumed to be present in them, since similar cases terminating in like manner, without the use of this remedy, must have occurred to most surgeons of experience,—still they cannot be repudiated as unworthy the notice of the practitioner, according to the law of evidence commonly received in physic. To remove all doubt, a much greater number of successful cases must be adduced, or an equal number of similar cases must be treated with and without belladonna, and the majority of favorable results have proved to be on the side of the treatment with this remedy. In respect of *hernia*, we must strongly protest against the adoption of any measures attended with loss of time and delay of the surgical operation, in a complaint of so urgent a nature as incarcerated hernia. When however, as sometimes occurs, through the strong opposition of the patient or his friends, an operation is impracticable, no mode of treatment which offers a chance of success should be neglected; and in such cases frictions with belladonna, harmless in themselves and soothing to the patient, are not only admissible, but are to be recommended, as supported by experience at least, if not by sound pathology.

Dr. Motard, of Turin, has found that a solution of belladonna, introduced into the nose, dilates the pupil effectually; and he is in the habit of moistening a pinch of snuff with a solution, by which means the pupil next to the nostril in which it is introduced is dilated in a minute or two. The dilatation lasts about two days. This hint is worthy of trial in those cases of cataract where the patients are in the constant habit of using belladonna to improve in some degree their imperfect vision; as it is a more convenient process than the common one.]—*Giornale delle Scienze Medico-Chirurgiche*, No. x. *Aprile*, 1835. —*British and Foreign Medical Review.*

4. *On the Influence of Atmospheric Heat in the Cure of Wounds and Ulcers.* By M. Jules Guyot, D. M. P.—[It is not necessary for us to make any remarks upon this memoir. Some of the facts that it contains are interesting, and their bearing upon surgical practice, not to mention other considerations, is sufficient to recommend them to the attention of other experimenters and to practitioners.]

Rabbits were selected as the subjects of experiment, on account of the delicacy of their organization, and the facility with which their wounds suppurate. Three different kinds of apparatus were employed; the first consisted of a wooden box, divided into four compartments, one above the other, and each capable of containing two rabbits.—These open behind by a slide, and in the front is a trough which contains the animal's food. The whole is traversed by a vertical tube, an aperture in which opens into each compartment, the size of which may be varied at will. Beneath the inferior extremity of this tube is placed a lamp, which burns constantly, and, the heated air passing through the tube, elevates every compartment to the requisite temperature. The degree of heat is ascertained by a thermometer passed into each compartment through an aperture in its side. A strong cloth is nailed over the front of this apparatus, with openings allowing the animals' heads to pass through them; so that the bodies of the rabbits remain in the heated air, whilst their heads are free in the external air, and they are able to eat from their troughs.

[As the experiments from which results of a practical character were derived were those made in this apparatus, it is unnecessary to describe the other two; one of which was for immersing an animal entirely in an uniformly elevated temperature, the other for the application of local heat.]

In the apparatus described rabbits can support, for days or weeks, a constant heat of from 113° to 147° Fahr., without sweat, without loss of appetite, or other disturbance than slight acceleration of respiration, which is not always observed, and which often ceases entirely. The same effects take place when the temperature is between 77° and 97° Fahr.; but without exception, when exposed to a heat between 97° and 113° Fahr., there has been extreme langor, with no appetite, and hurried respiration.

The following effects of heat thus applied on wounds have been observed:—Of four simple incisions, exposed constantly to a temperature of 140° Fahr., two united by the first intention, in from four to six hours; the other two remained gaping, and furnished during twelve hours a serous exudation, which gradually became dry, and formed over the surface of the wound a shining, rose-colored, thin, transparent varnish, which gradually cracked, whilst the wound diminished in extent. On the fourth day cicatrization was complete. This process had apparently taken place without inflammation; no suppuration occurred. Two incisions exposed to 122°, and two to 158° Fahr., united in a few hours, with the same phænomena as those just mentioned. Two incised wounds, exposed to a temperature of 86° Fahr., healed less readily; and it was only after the seventh day that cicatrization, which occurred without either inflammation or suppuration, appeared sufficiently solid to be exposed to the open air; one of these cicatrices continued sound; the other, in the course of twenty-four hours, had ulcerated and began to suppurate. Two incised wounds, exposed to the open air at 57° Fahr., remained twenty-four hours without any apparent change; the borders of the wounds then swelled and their surfaces furnished a considerable quantity of serosity.—They afterwards became covered by greyish yellow, soft, and opaque crusts. Towards the fifth day pus could be detected beneath, and on the sixth it escaped; the incrustations were then taken off, when the wounds presented a red and healthy appearance. On the seventh day a new crust was formed, and the wounds had contracted. The pus again collected, on the eighth day, beneath the crusts, which were again removed. On the ninth and tenth days, the borders of the wounds approached each other, and were covered with a linear incrustation, which fell on the twelfth day, leaving perfect cicatrices.

Elevated temperature was next applied to wounds in various conditions,—some nearly healed, others have incrusted; some suppurating; and four quite sanious, pale, and making no progress towards cicatrization. In these various states they were placed in the apparatus already described; two at 104°, and one at 122° Fahr. Three other wounds were renewed, either by removing their crusts or by making new incisions; one was placed in a heat of 86°, the other of 140° Fahr. Two more were similarly renewed, and in two other rabbits fresh wounds were made in the muscular substance of the thighs; after which they were all exposed to elevated heat. On the following day, the suppuration of one wound had much diminished; the four sanious wounds were red and almost dry; those which were renewed

8*

were covered by the varnish; the two new wounds still furnished a serous exudation. On the second day, the suppurating and four sanious wounds had incrusted. The rose-colored varnish was found upon the two fresh wounds, and over those which had been cleansed it remained without any suppuration during four days. During the night of the sixth day the lamp was extinguished. On the seventh, the thermometer being at 60° Fahr., the two cleansed and the two newly inflicted wounds were suppurating abundantly; the lamp was again lighted, the crusts removed, and the wounds cleansed. Three days were required to restore them to their previous state. By the fourteenth day of the immersion in the heated air no wounds remained uncicatrized.

At the suggestion of M. Magendie, M. Guyot was induced to try the effects of elevated temperature on the process of ulceration in man.

The apparatus employed is a box of an oblong shape, twelve inches long, and ten inches square at either end; closed at both extremities by a linen cloth nailed to the box, and having in it an aperture, through which the limb which is the subject of experiment may pass. This aperture is bordered by a running string, which allows of its being accurately fitted to the limb. The state of the wound is examined by a little door in the top of the apparatus. A tube is inserted into the bottom of the box horizontally, bent at a right angle; beneath the other extremity is a lamp. The degree of heat is regulated by a slide in the box, and estimated by a thermometer inserted into the same side. Those parts of the limb which are not contained in the box are supported by cushions; the whole is then fastened by tapes to the sides of the bed. The patient must observe perfect quietude.

Among the ulcers submitted to this mode of treatment was one of four years duration the sequel of a comminuted fracture, and which had resisted all the methods of treatment which had been employed; another, in an aged subject, old, painful, and indolent, which had occasionally cicatrized, but only for a short time; a third, of many years' duration, sanious, and covered with dark livid spots.

The effect of dry heat was favorable in all; in some it was continued until cure was effected. The ulcers became dry, inflammation and suppuration diminishing; pain was lessened, or entirely ceased; incrustation took place; the cicatrices were well formed, and showed no tendency to ulcerate.

The previous observations having been submitted to the Academy of Sciences, M. Roux was appointed to verify them by more experiments.

[We may select the following case from those which are recorded, as most strongly illustrating the effects of heat upon wounds.]

An ulcer of eight years' duration, occupying the whole of the posterior, inferior, and external part of the left leg, having an average diameter of four inches, was subjected to a temperature of 97° Fahr. The ulcer had been variously treated, and with some benefit, but had arrived at a state in which for three months it had made no favorable progress, under any means employed. The cicatrix surrounding it was thin and tense; its surface bled on the least touch, and the ulcer was situated over an enormous swelling of the fibula, and extended into a depression, which was owing to the loss of the tendo Achilles. The leg was deformed, and in such a condition that M. Roux conceived the only hope for the patient to consist in amputation of the limb. The constitution of the individual was extremely irritable, and he unwillingly submitted to the treatment. During the first two days suppuration was abundant, and the leg and foot swelled; on the third day they diminished, and some incrustation took place. During five weeks (the time that the treatment was continued,) the healing process went on, and at the end of this period the ulcer measured only ten lines by six. The cicatrization now stopped, and the wound bled on the slightest touch. The patient left the hospital, being unwilling to submit to amput tion, which was proposed to him.

The following conclusions are drawn by M. Guyot from his experiments:

1. Wounds have always healed more rapidly in a temperature above 85° Fahr., without dressing, than with or without dressing in a lower temperature.

2. Some wounds have healed in a heated atmosphere, which have not done so in one of the ordinary temperature.

3. In the former, the majority of wounds have healed without inflammation or suppuration; in the latter, this has not been observed.

4. Wounds have ceased to suppurate when exposed to heat, and have undergone the same healing process as fresh wounds.

From the effects of heat on man, it may be remarked that

1. An ulcer will heal, without any other means than the local application of increased temperature.

2. Heated air may give rise to the formation of a large cicatrix, in forty-eight hours, over an old ulcer.

3. In all cases, it aids, and constitutes one of the most favorable conditions for cicatrization.

4. Instead of giving rise to inflammation, it may check that which exists.

5. It acts to a certain degree on an internal malady.

6. It may be borne at 115° Fahr., during several weeks, without giving rise to serious accidents.

The employment of heat as a remedial agent, M. Guyot considers as evidently indicated in a certain number of cases; for instance, of local heat in wounds, ulcers, strumous engorgements, rheumatic pains, and white swellings; of diffuse heat (as by the first apparatus described,) in sciatica, accidental amenorrhœa, paraplegia; of general heat, in amenorrhœa of young women, the scrofulous diathesis, and phthisis. With regard to its beneficial operation in the last mentioned disease, it is asked whether it may not be inferred, from the marked improvement which has followed the exposure of ulcers on the external surface to increased temperature, whether a similar advantage might not be derived from its application to internal ulcers, through the medium of respiration?—*Archieves generals de Medicine*, 3me Serie, tome 8. *Juillet*, 1835.—*Ib.*

5. *Chlorine Gas as an Injection for the Cure of Hydrocele.* By M. Deconde.—Dr. Deblois, of Tournay, was in the habit of performing the radical cure of hydrocele by injecting chlorine gas instead of red wine. His premature death prevented him from making known the plan; but M. Deconde, who has seen its advantages, has described it. The chlorine gas is contained in a bladder, to which is fixed a pipe and stop-cock adapted to the canula of the trochar, into which it is fixed after the fluid has been evacuated; the stop-cock is then turned, and the bladder pressed so as to force the gas into the tunica vaginalis. When this is distended, the pipe and bladder are removed, and the thumb placed over the mouth of the trochar, so as to prevent the escape of the gas for the space of two minutes; it is then allowed to escape, and two or three repetitions of the injection are made, which are sufficient for the cure. The advantages are—the simplicity of the apparatus, and the whole sac being equally distended and exposed to the contract of the gas, which is not the case with fluid injections, which always gravitate. The danger also which sometimes occurs from the fluid being forced into the cellular tissue of the scrotum is avoided. M. Deconde proposes that the same remedy should be used in the cure of other diseases characterized by the secretion of serum in various cysts.—*Bulletin Medical Belge. Janvier*, 1836.—*Ib.*

6. *On Traumatic Ophthalmia.* By Dr. Schindler.—[Considering every fact as important which serves to illustrate the effects of injuries of the eye, we have perused with much satisfaction this paper by Dr. Schindler, whose name is already favorably known to British oculists, from his little work on Chronic Iritis after Keratonyxis.]

1. *From Burns.* The author first directs our attention to burns of the eye, and especially to burns from gunpowder. He remarks, that singeing the eyebrows and eyelashes, formation of blisters on the eyelids and their edges, more or less considerable vesications on the sclerotica and cornea, and grains of unignited powder fixed in the cornea, are the less serious effects of an explosion; while, in more severe cases, the destroying force manifests itself in laceration of the iris, separation of the iris from the choroid, laceration of the retina, or even destruction of the whole textures of the eyeball. Setting aside the severe cases in which the preservation of the organ is impossible, the bad consequences of burns of the eye are by no means in direct proportion to the extent of the injuries received. Considerable lacerations are sometimes followed by no permanent bad effects; while, in other cases, the mere dazzling of the eyes by the sudden flash of light, especially when the explosion takes place very close to the eyes, produces the most disastrous effects on the sight. Dr. Schindler is of opinion that, in the last mentioned cases, the over-stimulation produces an erethismus of the retina, passing into chronic inflammation and ultimate blindness. The patient, in one case particularly referred to, saw perfectly immediately after the explosion, and congratulated himself that his eyes were safe; but a speedy irritability to light, degenerating into severe photophobia, accompanied with slight external ophthalmia and considerable lacrymation, was the forerunner of an amaurosis erethica of the most dangerous description.

2. *From Slight Injuries.* It sometimes happens, after apparently trivial injuries of the eyeball, as a small cut of the cornea, a painful degree of pressure on the eye, the eye being struck by a bit of turf, or the like, that there immediately follows such a deprivation of sight, that the patient perceives objects only as if through a thick cloud, or that the eye even loses entirely its sensibility to light. There is no pain, and at first no appearance of inflammation. It is only after some days that a slight redness of the conjunctiva, and sometimes of the sclerotica, sets in. The pupil is black, but motionless, and either regularly or irregularly dilated. By and bye, the iris becomes inflamed, but the patient manifests no intolerance of light. Hypopium, if it occurs in such a case, may be removed; but the eye will, notwithstanding, always become atrophic, the cornea remaining quite clear, and only gradually shrinking. Mr. Lawrence refers such symptoms to laceration of the retina; but this opinion, grounded solely on the sudden loss of vision, does not appear sufficiently borne out. The nature of the injury scarcely admits, in some of the cases, of such an explanation, and seems to point rather to a paralysis of the ciliary nerves.

3. *From Severe Injuries.* Another and a very different form of traumatic ophthalmia arises from similar, but much more severe, injuries of the eye. Dr. Schindler has seen it follow a penetrating wound of the cornea or a blow with the fist. The patient, immediately after the injury, sees quite well, has no pain, and the redness of the conjunctiva is inconsiderable. The pupil is black and circular, not distorted, but slow in its motions. After some time, iritis comes on with its usual symptoms: the pupil, however, is at first considerably dilated, it loses its natural color, and even in a few days becomes of a fine sea-green, as in some cases of glaucoma. At the same time vision is extinguished. The cornea and the aqueous capsule do not in general suffer, so that the change behind the pupil is distinctly seen. Dr. Schindler considers it as impossible to decide whether that change depends on an alteration of the choroid pigment or of the vitreous humor. If, in the progress of the case, the cornea and its lining membrane should become nebulous, the change of color in the pupil can be perceived only so indistinctly that the observer will be at a loss whether to attribute it to cataract or to exudation. He will recognize his mistake, however, as soon as the cornea again clears. The iritis, in such cases running a subacute course, is attended with but little pain, and seldom produces any puriform effusion. After some weeks, the contracted pupil is closed by exudation, and of course all further observation of the deep-seated textures of the eye is prevented. This form also of traumatic ophthalmia always ends in atrophy, although Dr. Schindler acknowledges he long flattered himself that in such cases he might have preserved at least the form of the eye.

[We have frequently observed this form of traumatic ophthalmia. With regard to the green color which appears behind the pupil, and which bears so close a resemblance to glaucoma, it is plain that either glaucoma has been actually induced by the injury or subsequent inflammation, or a state similar in its effects to glaucoma. It is now well known that glaucoma is no opacity of the vitreous humor, nor reflection from an opaque retina, but that it depends chiefly on a change of color in the posterior lamellæ of the lens, by which these, having assumed a reddish brown or deep amber hue, absorb the violet and blue rays of the light entering the eye, leaving the yellow and green rays but little affected; whence results the green appearance of the humors in that disease.* Various substances in nature present, as is familiarly known, a different color, according as they are seen by reflection or by refraction;† and the glaucomatous lens is one of them. Seen in the eye, by reflected light, it is green; seen out of the eye, by refraction, it is of a deep amber or reddish brown. Now, in the traumatic ophthalmia in question, either the lens has undergone a similar change of color, as in glaucoma, or behind the lens there exists an effusion or deposition, productive of the same optical effect.]—*Zeitschrift fur die Ophthalmologie*, B. v. H. I. 1835. —*Ib.*

7. *Case of Cæsarean Operation performed three times successfully on the same Individual.* By Dr. Michaleis, of Kiel.—The patient was born in 1795 and during her childhood suffered from rickets to such a degree that she could scarcely walk alone till twelve years of age. The lower extremities, the spine, and pelvis, were much distorted. Her height is four feet three inches‡ the trunk is disproportionately long. On examination per vaginam, the antero-posterior diameter, from the lower edge of the symphisis pubis to the promontory, is about two and a half inches; from the upper edge of the symphisis pubis to the promontory, it is about two inches; the sacrum is nearly straight. Her first pregnancy was perfectly natural; the first pains having made their appearance, at the end of forty weeks, on the 16th of June, 1826. The os uteri dilated, and the membranes ruptured during the night. The Cæsarean operation was determined on the evening of the following day by the two medical men who had been summoned, but, as the necessary preparations could not be made immediately, it was deferred till the morning of the 18th; twelve leeches were applied to the lower part of the abdomen the night before, and some emulsion with nitre given to allay a feverish attack.

*First Operation.* On the morning of the 18th June, she was placed on a table nearly horizontally, on account of the abdomen being very pendulous. Dr. Seidel applied a napkin dipped in oil around the spot selected for the incision, and pressed the uterus, which was much inclined to one side, into the median line of the body, where he held it firm. The incision commenced in the linea alba, two inches below the umbilicus, and terminated two inches above the symphysis pubis. The divided integuments instantly separated a hand's breadth from each other. As the tension of the abdominal parietes was still so considerable as to prevent the finger being introduced, the peritoneum was divided by passing a director beneath, and cutting it with a blunt-ended bistoury. As soon as this was effected, Dr. Seidel pressed back some convolutions of intestine, which had protruded on both sides, so quickly that they produced no interruption to the operation.

When the uterine parietes were divided, the external wound required further dilatation before the child could be extracted. As the

---

*Philosophical Magazine, for June, 1832, page 470.

† Experiments and Considerations touching Colors. By the Rev. Robert Boyle. Third Part. Exp. 10 and 11. London, 1670.

‡ These numbers have been reduced to the English measure.

uterus contracted strongly, the placenta was immediately separated and brought away. A considerable hemorrhage from the edges of the uterus followed: cold water was poured upon it from a height; firm contraction followed, and the hemorrhage ceased. The child had been evidently dead some time. The lips of the wound came into such exact apposition that the suture was not deemed necessary, and they were merely held together with long strips of adhesive plaster; charpie and a common bandage completed the rest of the dressing, and the patient, who had been perfectly quiet during the whole operation, was placed in bed upon her back. A little cool drink was given her; a mixture of Decoct. altheæ, Potass. nitras, and Ol. olivæ, was ordered; and at night she took a grain of acetate of morphium. Pulse 120, small and hard. The night passed tranquilly; she slept well, slight perspiration, much thirst.

19th June. Feverish during the night. Urine passed without the catheter. The bowels not being open, she took every hour a table-spoonful of the following mixture:—℞. Decoct. Altheæ, ℥vj.; Ol. Amygd. dulc., Syrup. Rhæi, aa. ℥j. M.

21st June. Bowels have been moved seven times; pain in abdomen diminished; no fever. Half a grain of morphium was given several times daily until the 29th; she took in all one scruple.

23d June. The dressings were undone; the wound had united to the extent of three inches. The edges of the lower portion were not united, and were again brought together by fresh stips of adhesive plaster.

25th June. Felt very well; appetite good. Took Decoct. cinchonæ with camphor, and had some broth.

The only inconvenience of which the patient complained, and which continued to annoy her to the end of the month, was a scalding in passing water, which frequently produced retention of urine, requiring the catheter. An enema was necessary almost daily, to procure the necessary relief of the bowels. The lochia were regularly secreted; the milk was sparing.

In the course of three weeks the wounds was completely closed and cicatrized. On the 4th of July she sat up for the first time, and on the 10th she left her bed. The cicatrix was of about the breadth of a a finger; the abdomen was rather pendulous, and required a bandage. The catamenia returned in eight weeks after the operation.

Dr. Zwanck, the operator, attributed much of his success, and the remarkable absence of unfavorable symptoms, to the dressing having been made without suture, and to the free use of morphium.

*Second Pregnancy.* She menstruated for the last time on the 13th April, 1829. She quickened towards the end of August. She enjoyed good health during her pregnancy, except that the abdomen, being pendulous, hindered her somewhat in walking.

She was admitted into the Lying-in Hospital at Kiel, in December, 1829. External examination showed a strongly marked pendulous belly; this was evidently produced by actual dilatation of the uterus, and great distention of the abdominal parietes in the immediate vicinity. The cicatrix had now extended to the length of nine inches; it was four inches broad. The fundus uteri was high above the umbilicus: the distance of the umbilicus from the pubis was full fourteen inches, (fifteen English;) whereas, in her first confinement, it was stated to be eight and half, (nine English.) The uterus appeared to be in close contact with the abdominal parietes, and at the lower part of the cicatrix was evidently united with them for the space of an inch and a half.

In the beginning of January, 1830, she frequently complained of great distention of the abdominal integuments, at the lower part; the veins in the cicatrix were much dilated; and the little scars, where the leeches had bitten in her last pregnancy, now partially opened, and once during the night bled considerably.

*Second Operation.*—Pains commenced between the 20th and 21st of January. On the morning of the 21st, the os uteri was scarcely at all dilated; an extremity could be felt through the vaginia. By half-past four P. M., the os uteri was dilated about three fingers' breadth; the pains were strong, and membranes tense. As an extremity presented, and would necessarily prolapse in all probability with the cord, as soon as the membranes ruptured, it became no longer safe to delay the operation. Having therefore evacuated the bladder and rectum, it was commenced by the celebrated Wiedemann, of Kiel, in presence of Professors Luder and Deckmann, Dr. Michaelis, and several students. The two latter gentlemen supported the abdominal integuments with their hands. The incision, six inches in length, was made on the left side in the cicatrix, about an inch from the median line, where the child was felt most distinctly commencing at about five inches below the umbilicus, and terminating about three inches from the os pubis. The uterus was then divided layer by layer; a few vessels spouted for a moment. The placenta appeared in the wound, which was dilated full four inches. During a pain the placenta protruded, and occupied the opening. To separate the placenta on the right side, rupture the membranes, and extract the child (which had presented with a thigh) as far as the head, was the work of a few seconds. A powerful contraction of the uterus now retained the head for a few moments, and the child's life appeared in considerable danger; gentle traction soon released it, and the placenta followed. The operation lasted barely five minutes. The child, a girl, soon cried briskly, and the uterus contracted well.

The wound of the integuments was secured by three sutures, made after Grafe's plan, and supported with strips of adhesive plaster, and a small seton placed in the lower end. The patient, who had borne the whole operation without a murmur, complained of great pain in making the suture, and was very impatient.

After the operation the pulse was very slow and hard; her appearance was unchanged. On account of severe pains in the abdomen, (apparently after-pains,) twenty drops of Sp. Ætheris comp. and eighteen of Træ. Opii were given. She continually demanded morphium, which was not given. At eleven in the evening, as the pains were unabated, she had one grain of opium; at three-quarters past twelve she fell asleep, but was very restless from the after-pains.

Considering all circumstances, her recovery was favorable, so that, on the 21st of February, she was up for some time, and felt quite well. By the beginning of March the wound was entirely cicatrised, except a place or two in the skin. On careful examination, a fistulous opening was discovered, from which a little mucus could be squeezed. A probe which was introduced caused repeated bleeding, and at length penetrated downwards more than an inch into the uterus, which had become firmly united with the abdominal integuments. Injections into the fistula passed away immediately by the vagina, and a small quantity of muco-purulent fluid was constantly discharged from this passage. This fistulous canal resisted all attempts to make it heal until the patient left the hospital.

The union of the uterus with the abdominal integuments had so much increased this time, that, when the organ was fully contracted; it occupied a space of at least an inch and a half, and consisted therefore of its entire anterior surface. The child died on the 19th of February, of induration of the cellular tissue.

*Third Pregnancy.* The catamenia appeared in the course of a few weeks after her return, and the fistulous opening healed shortly afterwards of itself. In the middle of June, 1831, the catamenia appeared for the last time; and, at the beginning of March, 1832, she was again admitted into the Lying-in Hospital of Kiel. Her pregnancy had passed over without any peculiar inconvenience.

Early on the 28th of March pains made their appearance; by ten the same evening, the os uteri was transversely dilated to the extent of three fingers' breadth, and the membranes were very tense during a pain. The head could be distinctly felt above the pubis. The patient looked forward with confidence to the operation, but forbade most expressly the suture being made this time with Grafe's large needle.

*Third Operation.* Having evacuated the bladder and return, Dr. Michaelis commenced the operation in the presence of Professors Weidemann and Deckmann, etc.; the latter supported the abdominal parietes. The spot selected for the incision was to the left of the second cicatrix, where the child could be felt most distinctly. It was made about five inches long, in an oblique direction from above and outwards, downwards and inwards. On enlarging the incision into the uterus with a blunt-pointed bistoury, the liquor amnii escaped. An elbow presented, close behind which was the left knee; this latter was brought out; as, however, the nates did not follow gentle traction, the opening was dilated somewhat, and the child was extracted without difficulty. It was a boy, and cried immediately. The placenta was quickly separated, and brought away. Till this moment no fluid had escaped into the abdominal cavity, but now, as the uterus contracted imperfectly, a stream of blood issued from the wound; this was soon stopped by pouring water from a considerable height above it. No convolution of intestine protruded. The pulse was slow, not small. Some wine and Tinct. Opii were given her.

The healing of the wound advanced regularly until the 16th of May; there were some little places which had not cicatrised over, but these were quite closed in a few days more. On the 25th of May, Dr. Michaelis discovered another small opening in the lower third of the wound, leading through a fistulous canal into the uterus, which was firmly united to the abdominal integuments.

27th of May, the patient left the hospital with her child in perfect health. News was received of their good health on the 10th of June, and that the fistulous opening had healed. The child died sometime afterwards of scarlet fever.—*Neue Zeitschrift fur Geburtskunde*, B. iii. H. 3. 1835.—*Ib.*

8. *Connexion between Hypertrophy of the Heart and Apoplexy.*—The influence exercised over the brain by the heart, especially the left ventricle, is very great. Cases illustrative of this position, and the therapeutical views deduced from it, have been given, within a relatively short period, by Lallemand, Broussais, Andral, etc.; but in more fulness by M. M. Bricheteau, and Bouillaud, and Dr. Hope. Twenty-two cases of apoplexy, in all of which hypertrophy of the left ventricle of the heart existed to a greater or less degree, are narrated by M. Bricheteau. Among these is General Foy, the able and eloquent speaker, and one of the liberal leaders in the French Chamber of Deputies. From childhood he had been subject to palpitations of the heart. In 1817, when not quite forty years of age, he was threatened with apoplexy. This, as well as some subsequent attacks, were speedily relieved by copious venesection and other depletory measures, prescribed by Dr. Gall. The General was afterwards under the professional care of M. Broussais, who had known him in Italy, and who, aware that his patient had a tendency to heart disease, regulated his treatment accordingly, with great benefit to his general health. In 1823, he experienced an attack of enteritis and nephritis, and at this period the hypertrophy of the heart seemed to be much aggravated. In 1825, he fell down insensible in the street, but was soon recovered. In the November of this year, the cardiac disease proved fatal. There was found on dissection effusion to a considerable extent in the cavities of the pleura, and also within the pericardium. The heart was enormously enlarged in volume, and the walls of the left ventricle in particular were much increased in thickness. The head was not examined.

M. Bouillaud found, that, out of fifty-four cases of hypertrophy of the heart, there were eleven in which cerebral disease, in six apoplexy, and in five ramollissement or softening of the substance of the brain, was found on dissection. Dr. Hope, in a paper read (1835) before the College of Physicians, on the connexion of apoplexy and palsy with organic diseases of the heart, relates, that out of thirty-nine cases of apoplexy, disease of the heart was found coexistent in twenty-eight.

According to his data, the author concludes that the periods of life at which fatal apoplexy is most prevalent, are those in which disease of the heart (either hypertrophy of the muscular substance, or ossification of the valves and vessels) is of most frequent occurrence—namely, between forty and fifty, and between seventy and eighty. In some cases the existence of diseased and enlarged heart was not suspected. The deductions for practical guidance are clear—viz. avoidance of all severe bodily exercise, and all exciting emotions of the mind. And, as pointed out by M. Bricheteau, we should direct the occasional application of leeches over the region of the heart, instead of to the temples, or any other part of the head, the internal use of digitalis, hydriodate of potassa, and other diuretics*.—*Eclectic Journal of Medicine.*

9. *Frontal Neuralgia, thirty-two cases of.*—Dr. Rennes has published, in the *Archieves Generales* for June, some observations and re-

*Johnson's Medico-Chirurgical Review.

flections on thirty-two cases of frontal neuralgia, collected in the course of fifteen months, in private practice. The number, as Dr. Rennes properly remarks, is remarkably large; being thirty-two of neuralgia, out of four hundred of other diseases, or one to fifteen—in a rural district, (Bergerac.) Atmospherical vicissitudes and anomalies in the season, will, Dr. R. thinks, go far to explain the almost epidemic prevalence of the disease in question. Winter furnished the larger number, autumn and spring next; and summer the fewest. The medical constitution seemed to partake, during the whole of the period already mentioned, of a neuropathic character. Rheumatic pains were very common, and sometimes terminated in a well defined neuralgia. Facial neuralgia was seen to succeed gastralgia, and to alternate with sciatica. Sanguineous and nervous temperaments most predisposed to the disease, which in regard to age takes a wide range, being most frequent, however, in subjects between twenty and thirty-five years old. It seemed to Dr. R. to rarely depend on gastric disturbance; and he did not use, nor when used, did he derive much benefit from evacuants of the alimentary canal. Blood-letting was more commonly indicated, and was more evidently beneficial. He often had recourse to this remedy on the invasion of the disease, but it never cured even the persons who were relieved by it. The nervous element, as Dr. Rennes expresses himself, was too predominant. Two out of three cases were of females; and the greater number who invoked his assistance, were in a state of single-blessedness. Some of his younger female patients menstruated irregularly, and some were decidedly chlorotic. In one case, on the other hand, of a young lady, the neuralgia, which had preceded excessive menstrual discharge, causing anemia, was relieved after this latter. All classes of society, and all callings, seemed liable to the disease; although the poor were attacked in greater number. Of the house servants, cooks were the greatest sufferers.

Slightly noticing the symptomatology, which to the author exhibited nothing new, it is sufficient to say that the neuralgia was periodical in its attacks; returning each day at nearly the same hour; more commonly in the morning than in the evening. The type was always quotidian or double tertian; with the exception of one case, in which the tertian type was well marked. The intermittent form, less evident at the commencement, was well established in its progress. There was no uniformity in the duration of the paroxysm.

Dr. Rennes inclines to the opinion of those who, with the elder Frank, regard these neuralgias as true masked intermittents, *febres larvatæ*. In fact, during their prevalence, intermittent fevers, common in former years, were not met with. Confirmatory of this view, and the proof is that which most concerns us to know, since it is the practice pursued, it was found that, however, bleeding and opium or belladonna might modify or alleviate the malady, the cure depends on the administration of the sulphate of quinia. One or two days' use of this remedy, associated with narcotics, was sufficient for the removal of the disease.

Respecting emetics and purges, Dr. Rennes tells us, that he has not only failed to benefit his patients by their use, but he has found that their evacuating operation is prevented—rendered null by the neuralgia.

For allaying the pain during the paroxysm and diminishing the fluxion and weeping, which often accompany the frontal and palpebral neuralgia, the topical application of a strong solution of the extract of belladonna was found to be very serviceable. Compression of the nerve was never productive of any salutary result, whether by diminishing pain or otherwise. Cold application and galvanism were more useful, but only as palliatives.

We ought to return thanks to Dr. Rennes, both for imparting his experience, and for his modesty in being content, master as he was of thirty-two cases of neuralgia, in his own practice, to write a short essay in place of inflicting on us a large book. A London physician would not have allowed such an opportunity to escape of compiling a costly volume, and of either introducing or insinuating the superiority of his therapeutical means; perhaps claiming credit for originality in the use of the belladonna.—*Ib.*

10. *Paralysis cured by Electricity and Galvanism.*—In speaking of electricity and galvanism as two separate agents we conform with the general language of the day; although we are aware, that is now becoming the popular doctrine, which makes electricity, galvanism, magnetism, and caloric, modifications merely of one great principle.

Doctor G. Barrea relates the case of an Italian peasant woman who was seized with hemiplegia of the right side, soon after parturition—the power of motion being lost and sensibility diminished. He employed various remedies, both internally and externally, without any notable benefit; but a rougher medication, in the fashion of heavy thunder and lightening, the latter of which struck the house of the patient, frightened her out of her palsy so completely that on the following day she was quite restored, and continued well at the date of the account, written some months afterwards. There is an error in time in this history, whether on the part of the narrator or of the printer we cannot say. The woman was delivered on the 8th of July, 1835. About a fortnight afterwards, following some trouble in her sight, speech and intellect, she became paralytic on the right side, as already stated. The doctor was then called in, and administered and directed a variety of remedies, which, judging from the order of their enumeration, must have taken up some days. The paralysis he tells us still persisted, when, on the 16th July following, the curative incident, thunder and lightening, took place. Are we to suppose that the first date ought to be July 8th, 1834, in place of 1835? The material fact, however, remains the same; and should be added to other incidents, of a similar nature, of the curative powers of electricity and fright.

The other case is of a less questionable character in reference to the direct remedial agency of galvanism. It was of a young Polish officer who, in a charge on a battery, at the battle of Ostralenka, was thrown down, without any show of contusion on any part of his body, and deprived of all sensation. On coming to himself, he had lost his hearing, speech, and taste; at least all of this last which depended on the tongue. After being treated unsuccessfully at Vienna by bleeding and revulsives, and at Trieste by strychnia applied on the endermic plan, he went to Paris, and there M. Magendie had recourse to the action of galvanic currents in order to relieve his deafness. One of the wires of the pile was applied to the *chorda tympani.* At the very first application sensible effects were produced, and the patient experienced a strong humming sound. On the third trial, the sense of taste began to be restored; a curious fact to the anatomist and physiologist, by its throwing light upon the origin of the *chorda tympani* and the function of the fifth pair. After seven or eight applications of the galvanic current, the patient heard the sound of a drum, then of clocks, bells, and finally, speech. To complete the cure there is only wanting a restoration of the movements of the tongue. This it is hoped will be accomplished by the means already employed, only directing the extremity of the conducting wire on the laryngeal (lingual?) nerves.* It is, M. Magendie thinks, essential for the success of the plan, that there be direct contact between the nerve and the conducting wires—a condition easy of fulfilment in the case of the *chorda tympani,* the only nerve which is external in its course. In regard to the other nerves, with a little practice, they may be reached by a needle made to penetrate them in their course.

M. Roux related two cases corroborative of the importance of the recommendation of Magendie to establish a direct communication between the conducting wire and the nerve. The first was of a young girl, who had suffered from caries of the spine followed by paraplegia, and to whom, by means of galvanic currents directed to the spinal marrow along a conducting wire which touched this part, he completely restored motion. The second case was of inflammation of the facial nerve (*portio dura*) which was accompanied by a very disagreeable sensation in the tongue, but on the affected side only. M. Roux introduces this for the directly opposite inference to which it leads from the view taken by M. Magendie, in the restoration of the sense of taste after a stimulation of the *chorda tympani.—Ib.*

11. *Magnetism in Gout.*—The magnet, which some years ago was thought to work such marvels in the cure of rheumatism, and which is again lauded for the same end, has been spoken of recently for its curative power in gout. The matter is thus narrated in the *Bulletino delle Scienze Mediche* of Bologna. The editors of this work were led in consequence of a no small commercial demand for loadstone, to make inquiries concerning the uses to which it was put. It results from these, that the ex-dey of Algiers, whilst at Leghorn, in 1831, mentioned to a Catholic clergyman (Father Campagnati) who was suffering from the gout, that the application of the loadstone was an oriental remedy for the disease, and one of certain efficacy. The patient immediately procured a piece of loadstone, and applied it in the next paroxysm, which was entirely removed by it. Since then he has always had recourse to the same remedy, and he finds that the attacks come on less frequently and less severely, and that they invariably yield to the new treatment. On the first symptom he goes to bed, and places the loadstone in close contact with the painful part; he soon falls asleep, and awakes free from pain and able to walk. The loadstone he uses weighs five pounds and has smooth sides. He has been subject to the disease since 1805. Other gouty patients to

---

Nerfs larynges, in the original account.—*Archives Generales de Medicine,* Mai, 1836. p. 176.

whom he has recommended the remedy have experienced from it similar relief.—*Ib.*

12. *Proportion of Labors requiring Instruments.*—In confirmation of our preceding remarks, we shall give the testimony of Dr. Collins, who, for seven years, was chief physician, or master, as it is termed, to the Dublin Lying-in Hospital. During that period, 16,414 women gave birth to 16,654 children. Out of this large number of deliveries, instrumental assistance was required twenty-seven times only—the forceps twenty-four times, and the lever thrice. In thirteen of these twenty-seven cases, the labors were complex. The entire number gives us the average of about one in six hundred and eight deliveries. "According to this calculation," says Dr. Collins, "most physicians in private practice, should require to use the forceps or lever but very seldom; as, supposing an individual to attend four thousand cases in the course of his life--which is a greater number than falls to the lot of most men--instruments would be necessary in little more than *six* cases." The proportions given from the result of Dr. Joseph Clarke's experience, are 14 in 10,199.

The above estimate and remarks refer to the use of instruments with the intention of terminating a labor, when the uterus and its associate muscles are unable to accomplish the purpose; and always in the hope of preserving the life of the child, without detriment to the mother. They represent the ordinary course of things, provided what is happily the case in general, that the pelvis be well formed, and the uterus have no anomalous rigidity. But where there is a deformed or a too narrow pelvis, causing irremoveable impaction of the head of the child, or preventing its entrance into the pelvic strait, then another kind of instrument is used, and the case become a surgical one, in which it is necessary to remove the child, by first diminishing its bulk, as we would any foreign body, which by its location and presure endangers life. This is done by the crotchet. The use of the stethoscope enables the accoucheur to ascertain that respecting which he feels so much solicitude--viz: whether the child be still living, or is dead. If the latter, he has no hesitation in proceeding at once to its extraction. The proportion of cases of this description are much greater in Europe than in this country, and more in hospital than in private practice; for in Dublin many of the worst cases are brought to the hospital pending the labor, and after a discovery out of doors of their dangerous or desperate character. The result of Dr. Collin's observation shows that out of 16,654 birth, the perforator had been used, and the child destroyed in 118 of this number, or 1 in 141. Dr. Clarke's proportion, also deduced from hospital returns, was 1 in 208.

The following table shows the practice of different accoucheurs in reference to the use of instruments:—

| Physicians. | Total number of births | Forceps or vectis used in. | Crotchet used in. | Relative frequency of instrumental deliveries. |
|---|---|---|---|---|
| Dr. Carus of Dresden . . . | 2549 | 184 | 9 | 1 in 13 |
| Dr. Kluge of Berlin . . . | 1111 | 68 | 6 | 1 in 15 |
| Dr. Nægele of Heidelberg | 1711 | 55 | 5 | 1 in 28 |
| Dr. Boer of Vienna . . . . | 9589 | 35 | 13 | 1 in 199 |
| Mad. Boivin of Paris . . | 20517 | 96 | 16 | 1 in 183 |
| Dr. Merriman . . . . . . . | 2947 | 21 | 9 | 1 in 98 |
| Dr. Joseph Clarke . . . . | 10199 | 14 | 49 | 1 in 162 |
| Dr. Collins . . . . . . . . . | 16654 | 27 | 118 | 1 in 114 |

13. *Absorption of the Placenta.*—Due time being allowed for the contractile efforts of the uterus to detach and expel the placenta, and the desired result not being obtained, the accoucheur, as it is well known endeavors to procure the separation by suitable manual efforts, so made as not to either unduly irritate the uterus or give rise to inversion or other displacement of the organ. The dangers from allowing the placenta to remain adherent are uterine hæmorrhage in the first instance; and, should the uterus have contracted and all risk of this kind over, a fetid discharge per vaginam, retchings, abdominal tenderness and fever, until the remains of the placenta come away.—Another and different result and view have been lately suggested by a writer (M. *G. E. Maslieurat-Lagemard*) in the *Archives Generales* for May last. Agreeably to his showing, the adherent portion of the placenta is not broken down and gradually discharged with the lochiæ, but is actually absorbed, without any local irritation or notable constitutional disturbance. The female does well afterwards, and as a proof that the uterine functions are entirely restored and healthy, menstruation and pregnancy show themselves in due succession. Several cases occurring to different practitioners are recorded by M. Maslieurat-Lagemard, in such detail and in so authentic a guise as to render it difficult to deny the fact. Certain it is, that every accoucheur, of even limited range of practice, must have met with more than one case in which a portion of the placenta has remained behind adherent to the uterus, without his deeming it necessary to attempt its extraction from this latter, which has sufficiently contracted and been diminished in bulk to remove all fear of hæmorrhage. He must also be aware, that no inconvenience has resulted either at the time or subsequently in consequence of this adhesion.

A practical inference of some moment, from the view now taken, is, that the accoucheur should not, from a fear of the consequences of retained placenta, subject the female to irritating and protracted manual efforts for its removal, at the risk of inflaming the uterus and causing partial procidentia.—*Ib.*

14. *Prolapsus Uteri.*—Dr. Davis, in his *Principles and Practice of Obstetric Medicine*, recommends for incipient cases of descent of the uterus, active tonics, preceded or accompanied by alteratives; and where the vagina is in a relaxed state, astringent injections. When the prolapse is chronic, he considers the use of the pessary to be inevitable. The sponge pessary is the one which he prefers; but where it cannot be managed, the next best substitute is the ring pessary, made of light wood.

Dr. Hamilton (Practical Observations on Various Subjects relating to Midwifery, 1836 ) condemns the practice, so commonly recommended, of confining patients with prolapse of the womb to the horizontal posture, which is apt to injure the general health, and to increase the relaxation of the natural supports of the organ. He regards astringent injections as worse than useless; and he urges the following objections against the use of pessaries. They act only as palliatives; they irritate the vagina and keep up a mucous discharge; they make injurious pressure on the contents of the pelvis; they are apt to become incrusted with a calcareous matter, if not frequently taken out and cleaned ; and, lastly, "they subject the patient to a charge of the medical attendant for it."

The only instrument which Dr. Hamilton has used for a number of years past, is either a strong T bandage, or, in more serious degrees of the disease, a circular metallic belt like that of the common truss, provided with a cross or perpendicular strap, and to this strap is attached a cushion stuffed with horse-hair, about six inches in length by three in breadth, and of a thickness proportionate to the degree of relaxation, and therefore of the support required. "This bandage is to be worn whenever the patient is out of bed, as long as any symptom of the disease is perceived. It effectually relieves the unpleasant feelings, while it enables the patient to take walking exercise, which is so essentially necessary to the relief or cure of the disease." By this simple means, in conjunction with the use of cold bathing, and of appropriate constitutional treatment, Dr. H. has for many years past quite superseded the employment of pessaries.*

For many other details of practice and comparative observations in Obstetrics, we would refer to a highly instructive analytical Review on the subject, in Johnsons' Medico-Chirurgical, for July last.—*Ib.*

15. *Equilibrium of Population and Sustenance Demonstrated.*†—Dr. Loudon, in his original essay on this important subject, proposes a check both moral and healthful, to a redundant population; and he indicates also a dietetic principle, which, if carried into practice, would allay the fears of legislators respecting this redundancy.

The check may be expressed in a few words. It consists in prolonging the period of lactation, and by this means diminishing the number of births; since, as a general rule, mothers do not become pregnant during lactation.

For England, the question is thus presented. The increase of population is one per cent. annually. The average number of children to a marriage is 45. The average age of marrying is very near 24 years. Admitting then the time of marriage for females to be twenty-

* The reader who has seen Dr. Hull's abdominal supporter for the cure of prolapsus uteri, will recognize the sameness of principle, and in a great degree of instrument, as above recommended. The anterior pad over the pelvis in Dr. Hull's instrument, it is as liable to aid in misplacing, as to preserve the uterus *in situ*.

†The remainder of the title page of Dr. Loudon's work runs as follows: "*Showing, on Physiological and Statistical Grounds, the Means of obviating the fears of the late Mr. Malthus and his followers.* London, 1836."

four, and that of a child-bearing to terminate at forty-four, the average interval between each child will be fifty-four months, or four years and a half. Ten months is the common period of lactation at present. What increase of the time beyond this will be necessary to keep population in check, as it advances now in England? The reply is, about *fifteen months*, as thus:—To admit the nine months of gestation, the ten months of lactation, and one tenth of the remaining thirty-five months, as an equivalent for the present increase of population, which would make the period for suckling to be thirteen months and a half. But to this should be added six weeks, for the chances of impregnation taking place during the three months and six weeks; making the whole period as above, viz., fifteen months. The extension of lactation must necessarily increase the 4.5 years between each child to about 5, and consequently reduce the 4.5 children in a family to 4. Now, as one half die under the age of marriage for females, the result will be—only a representative for a father and one for a mother, on an average, in every family throughout the country.

The two points, one of medicine, and one of morals and economy, connected with this subject are, first: whether the mother's health will allow of her suckling her child for fifteen months; and secondly, whether the interval, if it were established, of five years between each child, would not leave a large number of the offspring unprovided for at a period when the parents were disabled by age from either seeing them set out in life or making some provision for their education and support. It would be a fortunate circumstance to the parties most directly interested, we mean the mothers themselves, if both the attention of them and of their medical advisers were more pointedly directed to the causes of imperfect lactation, and to the means of enabling them to avoid these and to go through the longer term with health and comfort. A more consistently rational diet, regular hours and suitable exercise in the open air, would be found, we believe, fully adequate to the emergency. Contrary course and practices are by no means confined, as we might at first suppose, in this country, to the rich, the fashionable, and the luxurious.

Dr. Loudon gives illustrations of his doctrine by pointing out the influence which the period of lactation exerts over the population of various countries.

The dietetic principle of this author, rather hinted at than fully laid down, would be to compare the amount of land susceptible of cultivation, and the nutritive properties, ascertained by experience, not conjectured by chemistry, of various articles of vegetable product, as potatoes, for example, with the population which they would support. Thus, he tells us that two millions and a half of acres of potatoes will permanently produce vegetable food for upwards of one hundred millions of people, or four times the present population of Great Britain and Ireland. There are, he says, seventy-six millions and a half of land in the United Kingdom, and thirty millions waste,—one half of which latter is capable of cultivation.—*Ib.*

16. *The Hydrated Peroxide or Tritoxide of Iron,—as an Antidote against Arsenic.*—The value of this preparation of iron, as a preventive of the effects of arsenic on the living body, has been tested in a favorable manner by MM. Leseur, Miguel and Soubeiran of Paris

Borelli and Demaria of Turin, in experiments on dogs; and by MM Geoffroy of Paris, and Bineau and Majeste of Sanmur, in trials on the human subject. Orfila, in communicating to the French Academy of Medicine the results of Lesur's experiments, thought that the German chemist, M. Bunzen, who discovered the antidote, had not overrated its importance.

In performing experiments to determine the effects of a poison on dogs, it must be borne in mind, that if a large dose, of arsenic for example, be given, and the animals allowed to vomit, it produced no effect. On the other hand, the operation to prevent the ejection of the contents of the stomach, which consists in tying the œsophagus, is itself fatal in its results. But as the arsenic kills in a few hours, if retained in the stomach, and the ligature of the œsophagus is not fatal often under two or three days, or even more, we can readily refer the death of the animal, into whose stomach arsenic has been introduced, and on which the ligature of the œsophagus has been practised, to its true cause. When, therefore, another substance is introduced at the same time with the arsenic, or soon after, and death does not take place for a relatively protracted period, we are authorized in believing in the counteracting agency or antidote operation of this substance, whatever it be. A dog, whose œsophagus was tied without any other change being induced, survived seventy-eight hours. Two other dogs, to which respectively nine and twelve grains of arsenic were administered, and which were prevented from vomiting by tying the œsophagus, died in two hours and two and a half. In the experiments subsequently made, the recently prepared peroxide of hydrated iron, mixed with water, was used in the proportion of twelve parts to one of white arsenic.

The results were that three dogs to which had been given poisonous doses of arsenic, to the one, twelve, and to the other two, eighteen grains each,—and subsequently, the hydrated peroxide of iron, the œsophagus being tied, survived the experiment,—the first, six days, the others, seventy-eight and eighty-four hours. From the first one the ligature was removed after twenty-four hours, but the deglutition of solids being impossible, the animal died on the sixth day.

Another series of experiments was instituted to ascertain how far the effects of the poison could be arrested by the iron, after some time had elapsed from the administration of the former. The results showed that in all, life was greatly prolonged by the iron beyond the time when it would have been destroyed by the arsenic. So far MM. Miguel and Soubeiran.

The experiments of the Italian physicians were still more conclusive. They need not be repeated here, except one, in which a dog was made to swallow ten grains of arsenic, and, immediately afterwards, three ounces of the hydrated peroxide; and its œsophagus was tied. The ligature was removed after twenty-four hours; and the dog continued to live for twelve days, at which time its deglutition was free; it was then killed by the same dose of arsenic as at first, but given without the peroxide of iron.

The case related by M. Geoffroy, of the antidotal powers of the iron, was of a hair-dresser, who, in a fit of delirium, tremens, took from his desk a paper which contained white arsenic, amounting, as was afterwards ascertained on inquiry, to at least a drachm and a half. In twenty minutes afterwards the hydrated tritoxide was obtained, and four or five pints of warm or cold water, charged with it, were given in a quarter of an hour—the treatment was continued for the next seven or eight hours, so that, altogether, the patient took twenty to twenty-five pounds of water, in which was suspended six ounces and four drachms of the preparation of iron. The first administration of the antidote was followed by copious vomiting and a stool, and during the remaining period the patient was vomited and purged three times. There was neither colic, heat in the throat, nor any symptom of poisoning; he complained of cramps in the fingers, and during the whole time was delirious, talking and gesticulating. He slept during the night ensuing the treatment, and in the morning appeared well.

The hydrated peroxide of iron used in the above experiments was procured in the following way. Sulphate of iron of commerce was mixed with five or six times its weight of water in a platinum or porcelain capsule, and when it boiled, small quantities of nitric acid were added until the ruddy vapor ceased to ascend; this was to bring the oxide of iron to its maximum of oxygenation. The liquor was then diluted, and the iron precipitated by liquid ammonia. The precipitate was washed and mixed with a small quantity of water until it had the consistence of clear "bouillie." As it cannot be weighed in this state, thirty-six times as much sulphate of iron is required as the poison taken, for the sulphate contains one-third of its weight of peroxide. It should be kept as a hydrate, for the dried powder does not act as an antidote. The chemical change consists in a conversion of the arsenious acid into arsenite of iron. Three times the quantity of the hydrated peroxide of iron is sufficient to neutralize arsenious acid in solution, and the decomposition is instantaneous; but as the arsenic when used as a poison is almost always swallowed as a powder, a much larger proportion of the antidote is advisable, for the action goes on very slowly. Even then, however, it must neutralize all ill effects, if it is in contact with the arsenic, for as soon as the smallest quantity of the poison is dissolved, the oxide of iron acts upon it and precipitates it.

It is recommended in all cases of poisoning by arsenic to give the patient large quantities of hydrate of the peroxide of iron; at the same time also to encourage vomiting, to get rid of undissolved arsenious acid from the stomach; and to repeat the hydrate as long as there are symptoms of any of the poison remaining.—*Ib.*

17. *Experiments on the Brain, Spinal Marrow, and Nerves.* By Professor Mayer, of Bonn.—Among the physiologists who have endeavored to investigate the functions of the brain and nervous system in modern times, Professor Mayer places Sir C. Bell foremost on the list; adding the names of Rolando, Belligeri, Magendie, and Flourens. The experiments of Flourens are some of the most important on this subject, but the great objection to them is the extensive injury which was unavoidably produced upon the brain, its nerves, and vessels, during these experiments; so that the precise effects upon the brain were probably more or less modified by the effects of the operations. Professor Mayer's object has been to repeat the experiments on a more simple plan; to avoid opening the cavity of the cranium, wounding the vessels, the severe hemorrhage, and exposure of the wounded parts to the atmospheric air; and thus to make the result more simple and certain. He has endeavored to determine how far the influence which the activity of the brain exerts upon the organic system is disturbed, where, without having undergone any injury, its supply of blood, which we must look upon as the chief source of its vitality and activity, has been partly or wholly cut off.

We may effect this object in two ways: viz. by tying the arteries which supply the brain, and by injecting some foreign fluid into these vessels. With regard to the first method, this chiefly refers to putting a ligature upon the two carotids, because tying the vertebral arteries at the same time is not only almost impossible to effect, but, as we shall also find, is instantly fatal. On the other hand, putting a ligature upon both carotids, and also upon one subclavian, may be effected, as will be seen shortly.

From numerous experiments of the kind just indicated. Dr. Mayer draws the following conclusions:

1st. That a healthy state of the cerebral activity is the necessary condition of life: in other words, the encephalum (viz. the brain, cerebellum, and medulla oblongata,) is the peculiar source of vital power,—the *fons vitalis*.

2d. The medulla spinalis, of itself, is not sufficient for the continuance of life, as its own life depends upon the vital energy of the encephalum.

3d. The vertigo and inability of preserving the upright posture is also a result of impeded cerebral activity. We are scarcely justified in attributing these effects to an injured state of the functions of the cerebellum, because, even in the experiments where the vertebral arteries are tied, the cerebellum receives a sufficient supply of blood.

4th. These experiments, moreover, tend to show that the brain directs and guides the vital functions. The cause or principle of the vital functions (viz. the circulation, respiration, nutrition, animal heat, etc.) is not in the encephalum, but the impulse to exert these functions of vegetable life emanate from it; and they cease when the encephalum sinks into inaction.

The physiological researches, especially during the last thirty years, both in this country and the continent, have satisfactorily proved that most, if not all, of the agents which exert such destructive energies on the nervous system do it through the medium of the circulation: this has been shown by the experiments of Christison and Coindet, of Brodie, Emmert, Viborg, and many others. Those of Sir B. Brodie on the action of the woorara poison are well known. Emmert showed this to be the case in a still more striking manner, by amputating the leg of an animal, and leaving it connected with the body only by means of the nerves; poisonous substances introduced into the foot produced no effects, not even when applied to the trunk of the nerve; and Viborg even applied one drachm of concentrated prussic acid to the brain of a horse which had been exposed by trepanning, without producing any effect. The experiments of Dupuy* on the contamination of the vital current strikingly confirm Dr. Mayer's observations. He found that injecting water, in which muscle had been soaked for four and a half years, produced symptoms in animals precisely similar to those of typhus; viz. debility, loss of sight, coma, falling of the head, etc. Gaspard† also injected mercury (half an ounce) into the carotid of a sheep, which produced insensibility, coma, and death in fifty minutes; but he does not appear to have carried the subject further in this direction, or to have made any practical deductions from it.

"The impulse," says Professor Mayer, "and the feeling of necessity to keep in action the vital functions, has its seat in the encephalum. Thus, when we reverse the experiment, and separate the head and brain from the trunk, especially in new-born animals, we observe symptoms of the above-mentioned impulse in the decapitated head. The heads of newly-born puppies or kittens, when thus separated from the body, suck the finger which is put into the mouth for ten or fifteen minutes. Attempts at respiration are made by opening the mouth, (a fact first noticed by Le Gallois,) and by the glottis alternately opening and shutting.

Dr. M's. fifth and last inference is, "that the above-mentioned experiments tend to prove, beyond doubt, that the circulation, the production of animal heat, and lastly nutrition and secretion, depend on the activity of the encephalum, and they stop when it stops; moreover, that the impulse to the continuance of these functions proceeds from the encephalum. It cannot be denied that a variety of causes connected with these functions have their seat in the body; but the main-spring, which sees all the wheels of the vital functions in motion, and without which they stop, resides in the encephalum."

Experiments have shown that the most vital part of the encephalum extends from the pons Varolii along the whole medulla oblongata, and at least as far as the second cervical nerve of the spinal marrow; in any part of which a wound is instantly fatal; that, as we descend along the medulla spinalis below this point, or ascend to the brain and cerebellum above it, the effects of injuries become gradually less fatal and dangerous. Professor Mayer's object therefore has been to subject this *centrum vitale* to a minute and rigorous examination, and he has shown it to be a rich field for observation and discovery.

The results of Professor Mayer's researches on the origin of the glosso-pharyngeal nerve, the par vagum, the hypoglossus, spinal accessory, and first cervical nerves, is to show that, of these, the hypoglossus, the spinal accessory, and first cervical, belong to that class of nerves which possess the double faculty of sensation and motion. The question is, does not this compound structure enter more deeply into the organization of the nervous system; or, in other words, shall we not find evidences of it in the phrenic, and even in the sympathetic nerves?

"With regard to the phrenic nerve," says M. Mayer, "I am aware of no researches in which its origin has been traced farther into the

*Injection des Matienes putrides dans la Veine Jugulaire du Cheval. Nouv. Bibl. Med. 1823, Jan., p. 90.

†Memoire Physiologique sur le Mercure. Magendie's Journal, t.i. No, 2, p. 165.

fourth cervical nerve. Fig. 1 not only shows the manner in which the phrenic gives off four twigs to the ganglion spinale of the fourth cervical nerve, but is also continued by means of two others along the anterior root of this nerve into the spinal column, so that we may justly say that the phrenic has its origin directly from the medulla spinalis. This is the more remarkable, because the phrenic, with the exception of its connexion with the cardiac nerves of the par vagum and sympathetic, is purely a muscular nerve, (going to the diaphram.) If we examine the sympathetic, we shall find that Soemmering has distinctly mentioned that it receives its roots, or connecting twigs, both from the posterior as also the anterior roots of the spinal nerves."

Professor Mayer has succeeded in tracing the supposed origin of the sympathetic into spinal marrow itself, not only in animals, but also in the human subject, and has given a drawing of the direct and indirect connexion of the sympathetic with the spinal marrow by the anterior and posterior roots of the second lumbar nerve. Fig. 2 shows it in the human subject; and fig. 3 in the calf, to the description of which he refers. According to this, he shows that the sympathetic not only communicates by means of many twigs with the ganglion spinale of the spinal nerves, and thus, with their posterior roots, but that one, two, or even three insulated twigs of the sympathetic are distinctly continued with those of the anterior root into spinal marrow.

"The nerves," says Professor M., "if I may so express myself, show a remarkable predilection for decussating or crossing. An arrangement of this kind I have observed in the cardiac nerves; the left branch passing to the right ventricle and pulmonary artery, the right to the left ventricle and aorta. This is not very distinct in the human subject; whereas, in animals it is much more so, and remarkably so in the horse"—"On the other hand, this arrangement of the nervous fibrillæ in the central parts of the nervous system,—viz. the brain and spinal marrow—is only observed at one spot, viz. at the origin of the corpora pyramidalia. I have always been able to detect this decussation in the human subject, although of variable extent; whereas, in many mammalia it does not exist at all; in others again, it appears."—*Ib.*

18. *Properties of Arteries leading to Inflamed Parts.*—Dr. Alison, of Edinburgh, has been led by various experiments to lay down the position, that the vital power of tonic contraction in arteries, called by Parry and others tonicity, and the only vital power existing in the arterial coats, is found to be *diminished* in all vessels leading to an inflamed part, of a size admitting of actual measurement. Taking the observations of this nature in connexion with those made with the microscope on the minute vessels of inflamed parts themselves, it may, the professor thinks, be laid down as a general fact, that all the vessels concerned in any local inflammation are in a state of *relaxation* and *distention*, as compared with their natural condition,—and this at a time when they are pulsating apparently with unusual force. These vessels transmit, in consequence of their weakened condition, the impulse given by the heart with less modification than it receives on passing through the arteries of sound parts: and themselves exerting a less power of contraction on the blood than they do in a sound state.

This being ascertained, the question immediatly presents itself, whether this weakened state of the vessels is an adequate cause of all the changes as to the movement of the blood which takes place in the inflamed state; that is, whether inflammation consists simply in a weakened action of the vessels. I formerly, says Dr. Allison, mentioned one striking fact, on which, I think, there can be no doubt, as sufficient to indicate that this question must be answered in the negative, viz., that two distinct and nearly opposite changes are ascertained to take place in the movement of blood through an inflamed part,—a retarded movement in the vessels most immediately concerned, and an accelerated movement and greatly increased transmission in all surrounding vessels.

The supposition that inflammation consists only in an altered action of the vessels, does not explain the effusion of lymph in this morbid process, nor show the difference between this state and simple determination or congestion of blood where no unusual product issues from the vessels. The peculiar products of inflammation exuding from the vessels are evidence that the fibrin is both in much larger quantity and has a *greater attraction* of aggregation than in any effusions from uninflamed vessels.

Irritants capable of exciting inflammation do not in their first action on the vessels produce the state noticed above; they *lessen* the calibre of the vessel, and *impede* the transmission of blood through them.

The more that this subject is considered, the more distinctly as I think, continues Dr. A., must it be perceived, that the only way to escape from these various difficulties is to suppose, that the causes which excite inflammation do so by really exciting or increasing a vital action, but not an action of the nature of contraction; that the idea of the vessels of inflamed parts taking on an increased action is a delusion; but what is truly excited is an action not *of the vessels* but *within the vessels* of the part affected; that it is an increased exertion of powers by which the blood is moved, or its motion influenced, in the capillaries, but which powers are inherent in the blood itself, dependent on, or influenced by, its relation to the surrounding textures, but independent of any contractions of living solids, and the increased exertion of which powers leads always to distention, and to more or less diminution of the tonic power of contraction, of the vessels, within which it takes place.—*Ib.*

19. *On the causes of the motion of the Blood in the Capillary Vessels.* By Dr. Poiseuille.—The globules of the blood in the capillaries of the mammiferæ are found to possess different velocities even in the same vessel. Some of them have two simultaneous motions—one of rotation, the other of translation; while others remain motionless for a time. The velocity of the globules in the capillaries is less than in the arteries and veins; or seldom greater.

Numerous experiments made, 1st, upon the heads of the salamander and frog, animals in which the circulation is, as it were, suspended at pleasure, show that it is established gradually from the centre to the circumference; 2d, upon the foot of the frog, dividing the crural vessels; 3d, upon the mesentery of the frog and the salamander, by cutting the heart; 4th, upon the mesentery of young rats and mice.

All these experiments, of which several are confirmed by those of the two celebrated physiologists, Haller and Spallanzani, have convinced Dr. Poiseuille that the heart and elasticity of the arterial coats are the *sole agents* in the capillary circulation in question.

In resting upon the preceding facts, that is to say, the action of the heart and arteries, and the tendency which the latter have to collapse when they are not sufficiently dilated by the tide of blood projected from the heart, the constant *jerking intermittent*, and *oscillatory* circulation, which precede the death of an animal, are easily explained; the cause of the *retrograde circulation* presented by the arteries after the death of the animal and that of the heart is similar.

Dr. P. has established, by a great number of experiments, that the calibre which the arteries and veins present, proceeds from the pressure of the liquid which they contain; that their coats are constantly distended by the blood which they receive; that these vessels tend to collapse suddenly, in consequence of the elasticity of their coats, as soon as the cause of their dilatation is removed.

The large arteries and veins, as well as the small ones, possess this property; but, besides, the diameter of the last gradually diminishes when they cease to receive blood. This retraction is sometimes so great, that the mesenteric vessels of the frog, salamander, young rats and mice, are reduced to two-thirds of their original diameter. He has also ascertained that, *cæteris paribus*, this reaction is more decided in the arteries than in the veins. These facts being known, it is easy to determine the motions of the blood in parts which have been separated from the trunk either by ligature or by a cutting instrument—motions which even yet are designated by the title of circulation.

Having cleared up these points, the author passes on to the examination of the causes of the irregular motions of the globules which he has observed in the capillary vessels.

If we study the course of the blood in the arteries and veins of the frog, of very young rats and of young mice, we observe, in proceeding from the axis of the vessel to the coats, that the velocity of the globules is totally different. In the centre, their velocity is at a maximum; it diminishes gradually as we approach the coats. In the immediate neighborhood of the coats, a very transparent space can be observed, which is generally occupied by serum; this space is equal to about the 1-8th or 1-10th of the diameter of the vessel. This transparent part of the vessels observed by Haller and Spallanzani as being occupied by serum, has been again noted by Blainville.

Since some of the globules, rubbing against each other, are projected into this transparent part of the vessels, the globules placed in the middle possess a very slow motion, and they cease to move when they are almost in contact with the coats of the vessel. The globules which are nearest to this transparent part have a double motion of rotation and translation; they roll, if the expression may be used, over this part of the serum.

From these observations, the author concludes that the interior of the vessels is lined with a layer of serum at rest. Since this layer is immoveable in its immediate contact with the coats of the vessels, every time that a globule is placed there it will be at rest, or rather, its velocity will be more or less diminished, according to the portion of the globule immersed in it. Now, in the capillaries the globules move between two layers of serum. Hence, their motion ought to be less rapid than in the large vessels, since they require to overcome the inertia of this layer.

If a globule is partly in the layer, this portion of the globule will be at rest, while its remainder, placed in the axis of the vessel, will acquire a certain verocity; then the globule will move round its own axis, in order to acquire its normal velocity in following the centre of the vessel. If of two globules, one is placed in advance of the other in the layer, the former will pursue its course, and the latter will be delayed, and the motions described will be presented.

The labors of M. Girard upon the flow of liquids in tubes of small diameter have established, in most tubes susceptible of being softened by the liquid moving in them, the existence of a similar layer. The author passed through tubes of very small diameter, liquids holding in suspension opaque bodies; and, having examined this current by microscope, he found this layer immoveable, and of a thickness much smaller than that obtained by the calculations of Girard.

Hence, the author concludes, that the blood transported by the vessel of the heart to all parts of the body does not impinge against the coats; that a layer of serum, by its state of rest, guards the coats from any such effects. Besides we can conceive the importance of this immoveable layer of serum lining the coats of the vessels in the act of nutrition, since the recent experiment of Muller of Berlin have demonstrated, that the fibrin is held in solution by the serum.

Dr. Poiseuille has farther studied the influence of cold and heat upon this layer of serum, and shows that the diminished velocity of the capillary circulation by cold and its greater rapidity by the action of cold, are naturally interpreted by the increase in the thickness of this layer in the first place, and its diminution in the second.

These results completely correspond with those of M. Girard on the variation in the thickness of the layer which lines the coats of inert tubes, when temperature increases or diminishes.

We know that certain animals, such as fishes, and some amphibious mammalia, are sometimes immersed nearly 262½ feet (80 metres) beneath the surface of the water, and then support a pressure of from seven to eight atmospheres. It is important, therefore, to know how this layer acts, and at the same time to observe the modifications of the capillary circulation under such pressure. With this object in view the author has constructed an apparatus, to which he has given the name of *Porte-objet pneumatique*.

From experiments made with this apparatus, he infers, that the thickness of the layer of serum, the existence of which is due to the affinity subsisting between the coats of the vessels and the serum, a thickness which varies so remarkably from heat and cold, is independent of surrounding pressure, that the contractions of the heart preserve their normal rythm, whatever the pressure is.

How absurd, then, is the opinion of these philosophers who consider that, without atmospheric pressure, circulation cannot go on; but atmospheric pressure, combined with the motions of respiration, are accessory causes of the flow of the blood, as Dr. Poiseuille has shown in another memoir.—*Ib.*

20. *Observations and Experiments upon the Functions of the Cœcum.* By Dr. Schultz, Professor of Physiology in the University of Berlin. The objects of the learned Professor in making his experiments were to ascertain; 1, the function of the cœcum; 2, the digestibility of the different articles of food; 3, the manner of the dissolution of the fleshy fibres in the stomach, according to microscopical observations; 4, the degree of acidity in the stomach and cœcum; 5, the degree of alkalescence of the food in the stomachs of ruminating animals; 6, the nature of the acids in the stomach; 7, the coagulation of milk by the saliva, stomach, etc.; 8, the saliva; 9, the nature of the bile.

He maintains that there are two digestions, one in the stomach, the other in the cæcum, and that the latter is more especially active when vegetable food has been ingested. From the first experiment he learned that the degree of acidity in the cœcum is not always the same, that it is not always present, and that the food may even become alkalescent. From the second, that this acidity was neutralized by long fasting, and thus allowed pure bile to enter the cœcum and neutralize its contents. From the third, that there is always bile in the course of the small intestines. From the fourth and fifth, that all the bile secreted by the liver during fasting, is by no means contained in the gall bladder, and that that part is very small compared with the large quantity that flows into the intestine during the empty state of the stomach. From the sixth, that though bile is always flowing, it never passes the cœcal valve during fasting, but collects on the upper side of it; it is only after perfect acidification, and at the beginning of the peristaltic motion of the intestines, that this bile flows into the cœcum. From the seventh, eight and ninth, that the degree of acidity and alkalescence of various parts of the digestive canal, vary with the length of time that has passed after feeding, and the degree of perfection of the gastric digestion, as also with the length of time which animals have fasted before feeding. From the tenth, that the quantity of digestible matter which is contained in the food has a great influence upon the degree of acidity in the cœcum. From the eleventh, that in carnivorous animals, when the cœcum and colon are but little developed, the food is for the most part digested by the stomach and small intestines, and the acidity in the cœcum is in general very weak, since the food, when it is here collected, contains little or no digestible matter.

The general results from the whole of the experiments we give in the author's own words; they are of the highest interest and importance.

"*Results of the experiments upon the cœcal digestion.*—It may, therefore, be gathered from my observations and experiments, that the food in the *cœcum* becomes not only a second time sour, but that the acid chyme is there neutralized by the access of bile, in the same way as in the *duodenum;* so that after the employing of the intestines very different reactions may be produced according to pleasure. On account of this twofold consumption of the bile in the stomach and cœcum, there is an antagonism between the two digestions; for when the bile is consumed by the digestion in the stomach, the cœcal digestion cannot be perfected, and, on the other hand, when the bile flows into the cœcum, the neutralization of the acidity in the duodenum cannot take place. In those animals in which the cœcal digestion is most perfectly developed, this antagonism appears to be so arranged, that each digestion has its particular period of action, so that when the one is in action, the other is either lessened or at rest. In ruminating animals, it is very evident that the gastric digestion takes place more particularly during the day, and the cœcal at night, so I think the gastric may very properly be called the diurnal, and the cœcal, nocturnal digestion.

"In carnivorous animals, however, the cœcum is so little developed, that the stomach alone furnishes nearly the whole process of digestion. These animals, therefore, have a preponderating diurnal digestion. This agrees with the fact, that carnivorus animals rest for the most part during the day, and at night become hungry, and seek their prey, and are, therefore, nocturnal animals, since their digestion takes place during the day.

"As the formation of the feces follows the perfected cœcal digestion, herbivorous animals are accustomed to discharge the greatest quantities in the morning and evening, and but very little during the day, and the healthy course of digestion. There appears to be something similar to this in man, in those ages where the cœcal digestion is most developed; in childhood, on the contrary, when the digestive apparatus resembles that of carnivorous animals, repeated discharges of excrement take place at indefinite periods of time.

"*The use of the valvula cœci in cœcal digestion.*—That the cœcal digestion may take place, it is necessary that the still digestible remains of the food should be rendered acid and changed into chyme, as in the stomach, before the mixture with the bile. This could not happen if the bile flowed continually into the cœcum, and it is therefore probable, that its opening into the small intestines is closed during chymification, as the stomach is closed during its digestion, only with the difference which the different state of the matter required. The stomach is closed during digestion at the pyloric orifice, to prevent the egress of the food, and the cœcum at its illiac opening, to prevent the ingress of the bile. This is my view of the use of the valvula cœci. I have not only found in general at the lower end of the *ileum*, an alkaline reaction, while the upper is still either sour or neutral, but at certain periods of digestion, a collection of pure bile at the iliac orifice of the cœcum. The contents of the cœcum are at this time nevertheless sour. This would be impossible if the mouth of the cœcum were not closed during chymification. After the collection of the food, therefore, in the cœcum, its opening like that of the bladder, uterus and stomach, appears to be strongly contracted by its muscular fibres, and with the help of the valvula cœci, to be perfectly closed. The contrary is the case at the beginning of the peristaltic motion of the cœcum, and upon the opening of its iliac orifice the collected bile flows in. This agrees with the sensation of the ceasing of the peristalic motion after the collection of the food in the cœcum, which I observed upon myself, and have described in my work (*de Alimentorum Concoctione Experimenta Nova.*) It appears to me, therefore, that the generally admitted explanation of Fallopius, according to which, the use of the valvula cœci is to prevent the return of the food from the cœcum into the *ileum*, is quite unfounded; for it may be easily seen that during excretion this backward motion is very possible.

The hygienic deductions from the above, made by Dr. Schultz, will be given in our next number.—*Ib.*

21. *Chronic Laryngitis.* By Dr. Roots.—This paper appears in the fourth Number of St. Thomas's Hospital Reports. A case is first narrated, and then follow the clinical remarks.

*Case* 1. J. Hurley, a coal-porter, aged 26 years, was admitted on the 24th December, affected for two months after exposure to cold, with severe cough, with thick yellow expectoration, succeeded by sore throat, and hoarseness. The tonsils were swelled, and the arches of the palate had a livid appearance. Pressure on the thyroid cartilage occasioned pain. The voice is heard in a hoarse whisper—the skin is hot and tongue coated, pulse 96. Bled to sixteen ozs.—blister to the throat—calomel purge. Antimonial wine and sulphate of magnesia every six hours.

28th. Can speak louder—has less pain on pressure—cough diminished—skin moist. 29. Twelve leeches to the throat—one grain of calomel, and a quarter of a grain of ant. tart. every four hours. Jan. 2d. Voice decidedly better; but there is still some pain on pressing the larynx. He continued to improve, and the mercury was discontinued on the 5th. On the 15th he was considered to be well, and was discharged on the 19th of January, 1836.

*Clin. Rem.*—The symptoms of chronic laryngitis are commonly these:—"A hoarse, harsh cough, occasionally almost amounting to a sort of croupy, or crowing sound; the voice, too, at the same time changes: it becomes hoarse, rough, and harsh, and as the disease advances, it is often merely a hoarse harsh whisper; sometimes, indeed, it is quite sibilant, accompanied by a hissing sound; and there is also a hissing sound sometimes in respiration. The respiration is difficult and hurried. The expectoration most commonly is copious; sometimes it is only mucus, but as the disease goes on, it becomes muco-purulent, sometimes almost entirely purulent, and sometimes sanguineous, either streaked with blood or thete is occasionally expectoration, with small patches of blood. The pulse is most commonly quick; and when the disease is of long standing, it is often accompanied by great emaciation, and attended also by a considerable anxiety of countenance. Sometimes the case is attended by a very distressing perspiration. There is also commonly more or less pain in deglutition, and if you put your hand on the larynx, on directing the patient to make an effort to swallow his saliva, you notice, so far as my experience goes, that it is invariably productive of pain to a greater or less extent. I never remember to have witnessed a case of chronic laryngitis, in which this symptom was not present.

"From the character of the expectoration, the emaciation, the quick pulse, and the perspiration, you will see that chronic inflammation of the larynx often simulates very closely phthisis pulmonalis. How then would you form your diagnosis? It is of the utmost importance that the diagnosis should be correct, inasmuch as the one is a case which admits of being cured, and the other, according to our present knowledge, does not. In the first place, you would judge by the degree of resonance on percussion being natural over every part of the chest, and more especially under the clavicles upon either side, the apex of the lung being the part in which, generally, tuberculous deposition commences. In addition, then, to the resonance being natural, you would, through the medium of auscultation, form your diagnosis by the state of the respiratory murmur. If the respiratory murmur was heard naturally over the whole of the chest, then you would arrive at the conclusion that the disease was situated merely in the larynx. It is often complicated, however, with bronchitis, and then you still find a good resonance, no dulness on percussion at any particular spot; but you have, to a greater or less extent over the large, and perhaps some of the small bronchial tubes, mucous, sonorous or sibilous rattle. This again is a condition which admits of being cured; and, therefore, you would make up your mind, under such a condition, that there was inflammation of the mucus tissue of the larynx, accompanied by inflammation of the mucus membrane of the bronchi."

These excellent remarks are followed by others of a similar character, respecting tuberculous deposits in the lungs, hepatization, excavations, etc. Chronic inflammation of the larynx produces, sometimes only a congestion of the lining membrane—sometimes thickening—hardening—roughness—tuberculation—ulceration. The ulceration may take place about rima glottidis, or even in the epiglottis—about the chordæ vocales, ventricles—the angle in front of the thyroid cartilage. Mere softening of the mucus membrane is a degeneration which Dr. R. has seldom seen.

The treatment of this complaint must be, to a certain extent, antiphlogistic. In many instances, it will be necessary to bleed once, twice, or even oftener, from the arm, besides frequent leechings. Counter-irritation, by leeches, antimony, or what Dr. R. prefers, croton oil, will be advantageous. Dr. R. warns the student against too much reliance on counter-irritation. Leeches must be again and again applied while there is pain on pressure of the larynx. Dr. R. prefers the croton oil to the tartar-emetic, especially in females and delicate subjects. The postules after antimony sometimes run much farther than is intended, and sloughs forms, which leave unseemly eschars on parts exposed to view, as the throat and nape of the neck.

In respect to internal medicines, he thinks the most efficacious is mercury, given in such moderate doses as to raise ptyalism in the course of a few weeks, rather than in a few days. Nauseants are of some use, in conjunction with mercury, and the best is antimony. A narcotic is generally necessary, in combination with the mercury and antimony, to allay the irritation of coughing. Opium is best, if the constipation be not troublesome—if so, hyoscyamus.

Where there is reason to suspect thickening of the mucus membrane, iodine may be employed, with the mercury. The recommendation of mercury in this disease is on the presumption, that there is no strong tendency to struma in the constitution. In our prognosis, we should always bear in mind that, sometimes, a sudden spasm of the glottis snaps the thread of life in a moment.—*Med. Chir. Review*, *October*, 1836.—*Ib.*

22. *Oil of Croton externally applied in Chronic Laryngitis.* By Dr. Romberg.—The following cases prove the peculiar efficacy of this species of counter-irritation in affections of the organs of voice; a fact observed by many.

Case 1. A fisherman, æt. 34, lost his voice after exerting himself greatly in saving some individuals from drowning. There was no reason to suspect any disorganization of the larynx. Blisters, vapor baths, etc., were tried without effect. Frictions of croton oil were directed over the larynx, to be repeated as soon as the eruption declined. On the twenty-first day of this treatment he began to recover his voice, and regained it completely.

Case 2. A girl, æt. 18, suffered during seven weeks with hoarseness, succeeded by aphonia, the consequence of a sudden chill.—Leeches, emetics, and irrritating frictions produced no relief; but, after the third application of croton oil, an eruption appeared, and she immediately regained her voice.

Case 3. A woman, æt. 38, complained for twelve months of a sensation of pressure in the pharynx, as if the neck were squeezed, rendering deglutition difficult; there were no other symptoms. Many remedies were tried without benefit. Three drops of croton oil were rubbed in, and, after the third application, an eruption appeared on the neck, nucha, chest and face, which was followed by erysipelas. The patient entirely recovered.

Dr. Romberg never found that the external application of croton oil had a purgative effect, but he never applied it to the abdominal integuments.

Dr. Otto reports, in the same journal, the case of a woman affected with sciatica, for which frictions with croton oil were made on the thigh, and the whole body became red and covered with vesicles. Dr. Otto never observed its purgative effect when thus applied.—*Wochenschrift fur die gesammte Heikunde.* 1835.—*Ib.*

23. *Diseases simulating acute inflammatory attacks of various important organs, and dependent on ganglionic or spinal irritation. The Edinburgh Medical and Surgical Journal,* for October, 1835, contains two instructive cases of this character, with some interesting remarks by John Torbet, Esq. These cases do not admit of much abridgment, and they are too long for insertion entire; but we shall present to our readers the substance of Mr. T's. observations.

*Neuralgia,* (a term which Mr. T. uses as synonymous with spinal, ganglionic, or cerebral *irritation,*) may attack any part of the nervous system, from the minute fibrils spread on the organs of sense, to the great nervous centres. "In attempting a general outline of such diseases," remarks Mr. T. "it is in vain to look for any thing like unvarying regularity of symptoms." We know nothing of the exact morbid condition giving rise to the symptoms. It may be of various kinds; and besides, in so delicate and complex a tissue as the nervous system, we may easily conceive the many modifying and controlling circumstances, that cannot fail to occur, in each individual case, when that system becomes the subject of morbid irritation, and thus vary the external phenomena. But though systematic exactness be out of the question, it is still possible to give something like a characteristic outline.

Of the diseases in question, females frequently before, sometimes after, puberty are the most usual victims. The individuals are of what is called irritable constitution,—a peculiarity, moreover, in some instances, distinctly hereditary, and connected more or less with strumous diathesis.

The morbid train of neuralgic symptoms is most usually set in operation either by a serious inflammatory or febrile disease, by excessive fatigue, or by injury, as from a heavy lift or severe fall. The severity and variety of diseases to which the individuals appear to be liable might almost characterize the affection. You are astonished to hear of their violent illnesses, the powerful remedies that are applied, and the rapidity with which they shake off these attacks. In severity, their diseases resemble those of no other person. They are paralytic, blind, speechless, without food, and without sleep for weeks or months, and yet they do not *die*.

Is the *head* the organ affected? They have head-ache, of which no words can convey an idea, ending perhaps in insensibility, or there is a heavy weight pressing down their eyelids, a tight bandage compresses, or hammers beat within their head, bells ring in their ears, vertigo and sickness overpower them, noise and light disturb them.

Is the *chest* affected? A sepulchral cough, each fit of which seems to threaten existence, over which medical art seems to possess no control, harasses from morning till night and night till morning. There is excruciating pain in the chest, especially during the cough, which is perfectly devoid of expectoration. There is a feeling of swelling and choking at the under part of the neck, tremendous palpitation of the heart; the pulse is quick and agitated, but varying greatly with the severity of the cough; and the heart's pulsations seem to occupy the whole left side of the chest.

Is the *digestive apparatus* the seat of disease? Then ensue vomiting without any apparent cause; incessant retching for hours, occurring perhaps every day for weeks, without any thing being vomited; no appetite; unquenchable thirst; occasional severe pain in the belly; or muscular cramps; and costiveness alternating with diarrhœa—the stools unaccountably copious.

Are the *organs of voluntary motion* the parts attacked? There are then cramps, prickling or numbness, sometimes severe spasms, liker tetanus or hydrophobia than any thing else, ending possibly in catalepsy, or paralysis of one or more limbs ensues.

Such is an epitome of the symptoms of the more violent cases; but an almost endless variety must of necessity occur, according as the several nervous centres are affected and their affections modified, by individual circumstances. It is precisely from this variety and anomaly of appearances that practical men are apt to be misled. The most marked symptoms are apparently so distant from, and unconnected with nervous irritation, and are moreover so violent and similar in their character to pure inflammatory diseases, that there is great risk of our chief curative efforts being directed to the appendages, and coincidents, instead of attacking the disease itself.

As to *diagnosis*, I shall confine myself to an attempt to distinguish neuralgic cases from cases of pure inflammation.

The constitution of the individual—the history of the attack, its frequently instantaneous seizure, its recurrence—the very severity of the symptoms, their occasional great discrepancy, their obstinate resistance to ordinary remedies, their metastatic and anomalous character, and apparent independence of organic affection—the minutely circumscribed locality which is the seat of pain or irregular action—the character of the pulse—the absence of the usual precursors of inflammation, or very abnormal character when present—are perhaps the chief circumstances to be attended to.

The constitution is extremely susceptible and irritable. In most cases it will be found that a variety of anomalous symptoms and affections, sick head-ache of great severity, retching, lancinating pains, langor and frequent faints, or aggravated hysterical symptoms, have been the precursors of a violent attack, although these may very likely not be considered as at all connected with the after train of symptoms. The attack, unlike inflammation, is not progressive, but in a very short time attains its height, and without shivering or other febrile precursors. The symptoms, though relieved for a time, are constantly recurring, and even often observe a degree of periodicity quite unlike inflammation. The pain in *neuralgia* is greatly more severe and agonizing than in pure inflammation, and is, moreover, not amenable to antiphlogistic or anodyne remedies. In inflammation there is no such phenomenon as excruciating pain darting from the back to the head, and instantly producing incoherence or delirium, nor such metastases, as from vomiting to cough, or pain in the chest to tooth-ache, or from cramp in the belly to overpowering head-ache with perfect relief to the alternate symptoms in the successive changes. In inflammatory diseases, if they continue a certain time, organic changes ensue. Vomiting or cough continuing for weeks, and dependent on inflammation, would unquestionably be accompanied with corresponding changes of structure in the respective viscera, more or less easily to be ascertained. The same symptoms continue for months in neuralgic cases without a shadow of evidence of any organic change. In neuralgic affections, (of the chest or belly for example,) the pain is confined to one spot, the least touch of which produces agony. This exquisite isolation is very rarely a character of inflammation. When, as sometimes happens, these internal and visceral pains alternate with similar acute pains in the limbs or external parts, the diagnosis becomes much easier.

In neuralgic cases, the firmness and hardness of the pure inflammatory pulse are not observed, unless in occasional combinations with the *phlegmasiæ*. On the contrary, along with great frequency, there is a bounding, a softness, and agitation in the strokes of the pulse, understood by practical men under the denomination of irritable pulse.

The violent symptoms in neuralgia are not ushered in with regular febrile precursors. More or fewer of these may indeed be present, but they want the irregularity they present in inflammatory attacks. In neuralgia every thing is in excess; the heat for instance intense, the thirst unquenchable, the pain extreme, but there is a discrepancy and hurry in the symptoms, which to say the least of it, forms the exception and not the rule in inflammatory cases.

When there is such discrepancy as violent pain, or incessant cough, with a calm and natural pulse; panting breathing without hurry of the circulation; sudden numbness or paralysis of the limbs occurring in the midst of a fit of coughing; violent head-ache or tooth-ache, or muscular spasm ending in catalepsy, etc., the nature of the case becomes very clear. Lastly, examination of the spine in many cases removes all doubt, by discovering to us points so tender as to cause agonizing pain, or perhaps the very symptoms of the disease on slight pressure. It must, however, be kept in mind that, if the ganglionic system be the centre affected, an examination of the spine affords no evidence of the morbid affection.

The prognosis in neuralgic cases, especially when not much complicated with inflammatory phenomena, may in general be favorable.

*Treatment.*—On this topic I need not dilate. When the nature of the case is clearly seen, there will I believe, be little difference of opinion as to the mode of treating it.

In complicated and violent cases, such as those narrated above, blood-letting to a greater or less extent seems absolutely necessary; but even in these cases this powerful remedy is to be viewed as a palliative not a radical cure. It may moderate the symptoms, and render their violence less likely to injure the various organs, but blood-letting will not remove the disease. On the contrary, I believe, a state of *anemia* greatly aggravates such distressing neuralgic affections, which in many of their symptoms greatly resemble that painful condition. In neuralgic cases, blood-letting will not calm the circulation, nor will it altogether remove or prevent the recurrence of pain; but judiciously applied, and apportioned to the strength of the patient, it will eminently palliate violent symptoms. In neuralgic cases blood-letting is to be resorted to as a palliative resource when other less energetic measures fail. In inflammatory diseases blood-letting is our chief dependence, and by its appropriate application we may fairly hope to remove the disease suddenly. While however, this important distinction is to be kept in view, we are also to recollect, that in neuralgic cases, inflammatory complications requiring more active treatment are occasionally recurring, but even here the neuralgic condition must influence our treatment.

Local blood-letting frequently affords temporary relief, and is generally safer than repeated general blood-letting. When the suffering point of the nervous system can be determined, however, topical bleeding there will afford infinitely more relief.

Detraction of blood in all forms, however, must be proportioned to the character and the violence of the symptoms, and the strength of the patient.

The judicious administration of purgatives is a matter of great importance; but so much has been written on this subject, that I deem it unnecessary to say a word.

To moderate the agonies of neuralgic suffering, *anodynes* must be a chief reliance. In many cases, indeed, they seem food and drink to the poor sufferer. In urgent cases they must be given in full doses, and frequently repeated, so as to keep up the narcotic effect. It is truly fortunate when opium in any shape agrees with the patient, for when it does so I consider it by far the best.

*Veratria.* I confess, much disappointed me. It produced the local tingling, characteristic of its action, but had not the slighest effect in relieving the painful symptoms, for the relief of which it was applied.

Of course in every case much modification both of the medicines and the form of their administration will be required; but after all it is to the suffering nervous centre that our chief attention ought to be directed.

When the ganglionic system is the centre affected, the symptoms are very uncontrollable, but even then, in addition to the general means already hinted at, local bleeding at different parts of the dorsal spine and chest, with occasional blisters or other counter-irritation, ought to be tried.

When from examination we have evidence that the spinal system is the diseased source, we have every reason, to be sanguine in our hopes of local treatment in the various forms in general use, and this part of the treatment ought to be our chief aim.

When the cerebral system is affected our applications must be directed there.

Absolute rest in the horizontal position is in many cases beneficial and necessary; free air and well regulated ventilation are very important adjuvants.

When the state of the patient admits of change of scene, country air, and gentle carriage exercise, they ought to be strenuously enjoined, as the benefits thus resulting are often great and striking.

The regulation of diet, and indeed many other particulars of treatment, Mr. T. purposely passes over, because they are obvious.—*American Journal.*

24. *The Atmosphere in relation to Malaria.*—That diseases, possessing a variety of types, originate from the existence of matter in the air we breathe, has been believed from the remotest ages. This is most distinctly true in woody or marshy countries, when attempts at colonization are first made. The fine town of Amaga, in Antioquia, Boussingault, (*Ann. de Chim.*, lvii. 150,) tells us, was founded in a forest. For six years, the population remained stationary; but, at last the roots and branches of the trees, having been completely removed in the lapse of a short time, the place became gradually more healthy, and it is now one of the most important little towns in the province. Panama, which was covered with wood, when the Spaniards first settled in it, possessed a deadly climate; but, at the present day, with the exception of the marshes of Chagres, the Isthmus is as healthy as any part of the coast of the Pacific in these latitudes. The influence of marshes in producing disease seems to be increased, when the water of the ocean becomes mingled with fresh water. Thus, Via Reggio, at the foot of the Appenines, in 1733, was so unhealthy, that the population did not exceed 330 inhabitants. But, in 1743, the sea-water was shut out from the lakes in the interior; and the consequence has been, that the town has become so salubrious, that the population, in 1823, amounted to 4000 souls. The town of Caitia, near Venezuela, from the same cause, is so unhealthy, that the negroes, when unable to pay their debts, made use of it as a sanctuary, well assured that their creditors will not have the temerity to pursue them thither. In such situations as we have described, where the climate is characterized by a high temperature and moist soil, the diseases of most common occurrence possess a highly putrid type, as yellow and intermittent fevers; but where the principal defect in the climate is the existence of great accumulations of vapor in the atmostphere, scurvy is the prevalent malady. Thus, in Choco, where rain constantly falls, it is an uncommon circumstance to meet with an individual who is not affected with this complaint.

On the elevated plains of the Andes, on the contrary, where the atmosphere is extremely dry, the inhabitants are subject to violent attacks of ophthalmia, as the children of the African desert.

In all marshy countries, the inhabitants recommend the same precautions, in order to prevent the accession of the endemic disease. From America to India, the traveler is directed carefully to avoid exposing himself to the evening dew.

Moscati took advantage of his knowledge of this fact, and endeavored to insulate the miasma dissolved in the air over the rice fields of Tuscany, by condensing it along with the dew. He observed in the condensed liquid small flocks, which possessed all the properties of animal matter, while the water, in the course of a few days, began to putrify.

In 1812, M. Rigaud de l'Isle made a series of experiments in the marshes of Languedoc with the same object in view. He condensed the dew upon glass. It exhibited the same appearances as that examined by Moscati; but, in addition, it afforded a precipitate with nitrate of silver, which rapidly assumed a purple color. But he endeavored to prove that such dew was hurtful to animals when taken internally, which was attempting too much, because it is sufficiently obvious, that cattle pasture on marshy herbage without suffering the slightest inconvenience.

In 1819, Boussingault remarked, while engaged in a geological excursion through the department of Ain, that sulphuric acid, when placed in the immediate vicinity of a marsh, became speedily black, while at a distance from the putrifying source it was not altered. At this period, fever was raging in the neighborhood, and, hence, he concluded, that the change in the acid was produced by the agency of organic matter, which existed in the air. Impressed with the idea, that sulphuric acid was a delicate test of the presence of organic matter in the atmosphere, on his departure to America, he consulted M. Humboldt, who approved highly of the suggestion. When in company with M. Rivero, he arrived on the banks of the lake Taricagua, the dry season had set in, the waters of the lake had greatly subsided; the ground, which had been inundated by the rains, was now emitting abundance of affluvia, and dire fever raged among the unfortunate natives. Thus was an excellent opportunity presented of ascertaining the accuracy of the opinion in reference to the agency by sulphuric acid. A quantity of very pure acid was, therefore, exposed to the air for 12 hours. At the termination of that period it had acquired a deep black tinge. No inference could, however, be deduced from this experiment, because the striking effect which he observed might be produced by the numerous insects which swarmed in the air.

In 1829, he operated in a different manner. He was then at Cartago, in the valley of the river Cauca, which in its sluggish course gives origin to several Lagoons, from whence during the prevalence of the south wind, the malaria is propelled to the town, and produces abundance of disease: such was the state of matters when Boussingault visited the place. A little after sunset, he placed two watch glasses on a table in the middle of a marshy meadow. Into one of the glasses he poured warm distilled water, in order to moisten its surface, and to raise its temperature above that of the air. The other glass being cold was speedily covered with dew, the warm glass received no condensed liquid. When a drop of distilled sulphuric acid was added to each glass and evaporated by the heat of a spirit lamp, a trace of carbonaceous matter was always detected in the dew glass, while none was observable after volatilizing the acid alone. He repeated his experiments, during several evenings, but was then laid on a bed of sickness, by the agency of this very substance which he was endeavoring to detect. These trials showed the presence of organic matter; the next object was to ascertain its quantity. Admitting that miasma as other organic substances contained hydrogen, he considered that if the quantity of this gas were appreciated, the great step would be gained. For this purpose, in 1830, he passed a given weight of impure marshy air through a red hot glass tube, and collected the water formed in a tube filled with chloride of calcium. A quantity of air weighing from 4697 grs. to 4774 grs. in different trials, afforded .77 gr. water, which is equivalent to .077 gr. hydrogen. When the air, previous to being passed through the hot tube, was brought in contact with sulphuric acid, it yielded no organic matter. Hence, he concludes, that it is very probable, that the malaria is a flocy matter, and that the employment of a veil as a precaution against the influence of malaria may be efficacious.

The preceding experiments, did not apparently indicate the presence of hydrogen, when it had been previously washed with sulphuric acid. To determine this precisely, it was necessary to experiment with great nicety. A vessel with sulphuric acid was made to communicate on one side, with a gasometer filled with common air, and on the other led into a tube 8 or 10 feet long, filled with chloride of calcium. To the latter, was joined a glass tube filled with asbestus, moistened by sulphuric acid; a tube filled with copper turnings and passing through a furnace followed next, and conducted the air into another tube filled with asbestus, soaked in sulphuric acid. In the last tube the water was condensed, when any hydrogen was contained in the air submitted to experiment.

| | | | | |
|---|---|---|---|---|
| From | 1609.6 grs. | were obtained waters | =.0172 | gr. hydrogen. |
| " | 5482.4 " | " | =.0443 | " |
| " | 6498.8 " | " | =.0477 | " |

The proportion of hydrogen formed in the air on different days is represented in the following table:

*Hydrogen in one part of Air.*

| | | Weight. | Volume. |
|---|---|---|---|
| 2nd and 3d | April, 1834, | 0.000008 | 0.00013 |
| 4th and 5th | " | 0.000007 | 0.00012 |
| 23d | " | 0.000002 | 0.00004 |
| 28th | " | 0.000005 | 0.00008 |
| 31st May, | " | 0.000003 | 0.00005 |

Boussingault, having thus, as he conceives established the fact, (although this inference might be considered premature) proceeds to speculate upon the nature of the compound, of which the hydrogen forms a constituent. He considers it to be in union with carbon, forming carburetted hydrogen.

Saussure inferred from his experiments that an inflammable gas existed in the atmosphere with a base of carbon. Boussingault ascribes its origin to exhalation from the earth, in the decomposition of vegetable matter, and also from mineral sources.

Near the falls of Niagara there is a fertile source of it in the *Burning Spring*. In Italy and Sicily it occurs in abundance, and in China, about Kratnig-fou there are within 50 square leagues, no less than 10,000 salt pits, from all of which, inflammable gas is emitted. Near the town of Kioung-tcheou, there is a pit of 5½ feet deep and 16 in diameter. The gas proceeding from this pit, when set on fire, forms such a blaze that the whole country is illuminated during the night to a great extent. Near Bakow, likewise, according to Imbert, there are some extensive sources. Discharges of the same nature frequently occur in this country. Dr. Thomas Thompson has described one, which existed in the neighborhood of Glasgow, where the gas burst forth in different parts of a hill side. When examined it was found to contain only 12 per cent. of common air.—*Records of General Science*, Sept. 1836.—*Ib.*

25. *On the Physiology of Vomiting; and on the Causes of its Difference in Adults and Children.* By Professor C. H. Schultz, M. D.—The great frequency of vomiting in infants at the breast, and the spontaneousness and facility with which this process takes place, are well known. It seems to occur without any previous nausea, as the infants, generally speaking exhibit no signs of uneasiness. The case, as is well known, is very different with adults, in whom nausea and retching will, in certain cases, exist in a great degree for days, or even weeks, without any evacuation of the contents of the stomach. The facility of vomiting in general remains with children for some years after weaning, although this is effected with somewhat greater difficulty than during the period of nursing. The causes of this difference in the readiness to vomit at different ages has not, as far as I know, been yet closely investigated.

To enable us to prosecute this inquiry with advantage, it is necessary that we should have a perfect understanding of the causes of vomiting in general; and to this point I shall address myself in the first place.

The opinion first advanced by Boyle, that, in the act of vomiting, the stomach is passive,—the evacuation of its contents being effected by the contemporaneous contraction of the abdominal muscles and diaphragm,—has been adopted and powerfully advocated by physiologists of the greatest name, more especially of late years. Chirac confirmed the fact stated by Boyle, that no convulsive motions are felt in the stomach during vomiting in the case of dogs, when the hand is placed in contact with the organ through a wound made in the abdomen. Van Swieten, Senac, and others, adopted the opinion of Boyle on the grounds; and, in later times, Magendie has proved beyond question, that, in the case of dogs, not only are no convulsive motions of the stomach *felt* during vomiting, but none are *seen* when the stomach is laid bare; and, moreover, that when the abdominal muscles are removed, and the contractile power of the diaphragm destroyed, the act of vomiting in dogs, if not entirely prevented, is, at least, rendered extremely difficult. It accords with this view of the process that, in man, vomiting becomes easier in proportion as the stomach is distended, and is thus more exposed to compression between the above-named muscles.

The objection to this explanation, derived from the fact that vomiting takes place in birds and amphibia which have no diaphragm, as also in certain cases in the human subject in which an abnormal position of the stomach had removed it from the pressure of this muscle, is not valid, since in such cases the thoracic viscera, during inspiration, present sufficient resistance to allow the stomach to be compressed between them and the abdominal muscles. It is indeed obvious, that the same muscular action takes place in the act of vomiting as in labor, cough, and the evacuation of the bowels and bladder, etc.; and that the discharge of the contents of the stomach by repeated fits or impulses, corresponds exactly with the spasm-like contractions of the abdominal muscles and diaphragm.

It has not, however, escaped the opposers of Magendie's theory, that if vomiting were effected exclusively by the abdominal muscles and diaphragm, it ought to be a purely voluntary act; whereas, it is known that only very few animals, such as frogs and birds of prey, can evacuate the contents of the stomach at pleasure. It results from this fact alone, that the before-mentioned muscles are not exclusively those which are active during vomiting; and we are hence led back to the old doctrine of the anti-peristaltic motion of the digestive organs. Maignault and Beclard have attempted to prove that, although the stomach is not spasmodically contracted, still that the œsophagus is thus affected, by fits, during vomiting in the dog; and every one who has experienced vomiting in his own person must have felt that these reverse spasmodic efforts of the muscles of deglutition commence in the pharynx. These gentlemen were further of opinion that, in the act of vomiting, no antiperistaltic movements take place in the stomach, but that this organ presents a state of equable tonic contraction, and that it is only by means of the fitful contractions and expansions of the œsophagus, aided by the action of the abdominal muscles, that the stomach is emptied of its contents.

While acknowledging our obligations to the French investigators, we must admit that there are many phenomena attending the act of vomiting which prove their theory to be at least insufficient. If the œsophagus and abdominal muscles are the only parts active during vomiting, how is the phenomena of fæcal vomiting to be explained? I consider this morbid state sufficient proof in itself that an antiperistaltic action both of the intestinal canal and stomach does exist, while, on the other hand, no one can deny that there may and do exist contractions of the abdominal muscles, diaphragm, and œsophagus, without any vomiting. This is evident in the case of the horse, rabbit, hare, guinea-pig, and several other herbivorous animals, which cannot be made to vomit even by the strongest emetics, although the strongest retching and contractions of the abdominal muscles take place, and although they possess the same organs as the dog, which vomits on the slightest occasion. It is the more important to investigate the cause of this difference in animals, as it will lead to the explanation of the much greater facility of vomiting in children than in adults.

The cause of these differences lies in the particular shape of the stomach in different animals, a circumstance, as far as I know, hitherto unnoticed by comparative anatomists; and the same cause operates in producing the difference in the facility of vomiting in the infant and the adult; since there exists the same analogous difference of form between the stomach of the child and the adult man, as between the stomach of animals which vomit with facility, such as the dog and cat (and we may say carnivorous animals in general,) and the stomach of those which vomit not at all or with extreme difficulty, as the horse and rabbit, (and herbivorous animals generally.)

Before proceeding further in the enquiry, I think it necessary to state that my experiments and observations lead me to decide positively in favor of the existence of antiperistaltic motions of the stomach during the act of vomiting. Boyle, Chirac, and the recent observers in France, hastily concluded that, because they could discover no *convulsive* movements of the stomach that therefore there were no antiperistaltic movements of any kind: they found the stomach contracted and motionless. I admit that there are no convulsive movements, but I cannot concede that in the dog, for instance, the stomach is at rest during the act of vomiting. On the contrary, I maintain that decided antiperistaltic movements are perceptible, but these are not stronger than the ordinary peristaltic motions of the same organ. They are, moreover, not very distinct in the middle portion and fundus of the stomach, but only at the two extremities near the cardia and pylorus. The whole pyloric portion is strongly contracted when the cardiac portion expands; and, while this is going on, there is no perceptible motion in the fundus and larger curvature, and assuredly no convulsive one. But, it may be asked, what considerable effect can so slow an anti-peristaltic motion have in vomiting? The answer is briefly this,—that, by this anti-peristaltic motion, (no doubt assisted by the abdominal muscles,) the *direction* is given to the food which is to be ejected by the act of vomiting, or which is to be forced from the intestines into the stomach in the case of fæcal vomiting. If the abdominal muscles alone acted on the perfectly passive stomach, the food might, by this pressure, be driven into the intestine as well as into the œsophagus; if, then, the contents of the stomach are to be ejected in a particular direction, it is requisite that the cardiac and pyloric portions should possess a distinct active motion.

I now return to the various forms of the stomach occasioning the differences in vomiting; and here I may take for granted as understood what I have detailed in the work, "*De Alimentorum Concoctione*," concerning the forms of the stomachs of carnivorous and herbivorous animals. It is demonstrable that a child's stomach is as different from that of an adult as a pole-cat's is from that of a rat; and, if the difference between the form of a child's stomach and that of an adult has not been sooner recognised, it is only because their very different functions and importance in the preservation of life had not previously been suspected; for this difference will not fail to strke every one as soon as his attention is directed to it. But, to make these differences still more conspicuous, I will introduce an outline of the form of a child's stomach, and that of an adult.

*Fig* 1.

The stomach of a child (*Fig*. 1.) is more of a conical form, drawn out lengthwise, and gradually narrowing towards the two extremities, inferiorly towards the pylorous (*b*), superiorly towards the cardia (*a*). The œsophagus is inserted into the fundus at the left extremity, and at a distance from the pylorus; the small curvature is stretched out lengthwise (*c*), the large curvature (*dd*) is less developed, and runs almost parallel with the small; in short, the stomach of a child resembles that of the carnivorous mammalia.

*Fig*. 2.

The form of the stomach of the adult is very different (*Fig* 2): it is more circular; the œsophagus (*a*) is not inserted into the left extremity, as is the case with the child's, but into the middle between the left extremity and the pylorus (*b*). The pylorus itself is drawn back towards the cardia, and both brought very near to each other; on this account, the small curvature is very short (*ce*,) while the large curvature, on the contrary, is disproportionately extended (*dddd*,) forming not only the entire lower circumference of the stomach, but also surrounding that part of the fundus situated between the cardia and the left extremity; so that the large curvature alone forms about four-fifths of the whole circumference of the stomach. It must also be added, that the fundus does not pass into the pyloric portion gradually and gently, as is the case with the child's, but that the latter is separated from the former by a sort of neck or contraction (*ce*,) sometimes more, sometimes less, strongly marked. In consequence of this the left part of the stomach assumes an almost circular form, and the whole very much resembles the form of the stomach of the rat or rabbit, although in a less marked degree than in these animals.

To each of these different forms of the stomach, an entirely distinct motion, peristaltic as well as anti-peristaltic, has been given. In the child's stomach, where the small curvature is extended almost parallel with the large one, the food is expelled with nearly equal power by the undulating motion of both curvatures, and forced towards the pylorus by the peristaltic and towards the cardia and œsophagus by the anti-peristaltic. In consequence of this, vomiting in children is very easy, because the œsophagus is situated at one extremity of the stomach, towards which the food is forced, at the same time that the pylorus closes and the cardia opens. But the process is very different in the stomach of the adult; in this, the small curvature is so much shortened, and the large one so much extended, that the food is not equally propelled from both sides, but the motion is almost confined to one side, and is effected principally by the large curvature, which embraces almost the entire circumference of the contents of the stomach; by this partial action, the contents of the stomach are moved rather in a rotary direction, which completely stops towards the contracted pyloric portion, turning round in the fundus from the left side to the right when urged by the peristaltic motion, and from the right to the left when by the anti-peristaltic. In consequence of this, during the act of vomiting, the anti-peristaltic motion does not direct the food towards the cardia and œsophagus, but merely communicates to it a motion contrary to that given by the peristaltic; and herein the reason is to be sought why, notwithstanding the pressure of the abdominal muscles and the diaphragm, the contents of the stomach are so difficult to be voided, and that, in many herbivorous animals, where the small curvature is still more shortened, the evacuation of the contents of the stomach of an adult can be effected only by a strenuous effort, produced by the strong pressure of the diaphragm and abdominal muscles at the same time that the œsophagus opens and shuts alternately; the stomach itself would be incapable from its anti-peristaltic motion alone to discharge its contents upwards. In this respect there exists a completely different state of things in the pyloric and cardiac portions of the stomach. The pyloric portion from the point where it is so much reduced in diameter, exhibits a more regular or intestine-like form of both curvatures, and the contents are on that account easily urged forwards into the duodenum; but, in the other direction, the contemporaneous motion of the two sides ceases beyond the contracted part, becoming, as already stated, rotary, in the cardial portion.

These details satisfactorily explain the differences so often referred to between children and adults. The former can discharge the contents of their stomachs by the anti-peristaltic motion alone, without any perceptible assistance of the abdominal muscles; and the least pressure from these will increase the discharge. Animals whose stomachs are cylindrical, and in which, consequently, the ordinary relation between two curvatures entirely ceases, such as frogs or fishes, can, as it appears, with facility empty their stomachs by means of the anti-peristaltic motion alone, without any co-operation of the abdominal muscles; and it is thus that they often throw up pieces of food merely on account of their inconvenient position in the stomach, and swallow them again in a more acceptable direction; even dogs after having swallowed a piece of bone frequently adopt a somewhat similar method. The human stomach in the earlier stages of its formation puts on the cylindrical form of the stomach of fishes and amphibious animals; in the embryo it appears only as a slight enlargement and elongation of the œsophagus in the abdominal cavity, with the cardia directed upwards and the pylorus downwards, as is the case with frogs. The stomach assumes its horizontal position only at a later period when the curvatures become developed.

There are naturally an endless number of transitions and intermediate stages of development, between the cylindrical, conical form of the stomach of the infant and that of the adult; and these numerous transitions will be accompanied by as many degrees of facility or difficulty in vomiting. What appears to me particularly interesting in a medical point of view is, that the round stomach of the adult is frequently seen in children of a diseased or merely of a disordered condition at a much earlier age than usual, and that such children also generally vomit with much more difficulty. I have had opportunities of making this observation in several postmortem examinations of scrofulous children; and in one instance was able to describe before death the probable form of the stomach, from the extraordinary difficulty with which the child vomited. On the other hand, the fundus of the stomach of adults is not always found to extend, in a like degree, beyond the insertion of the œsophagus towards the left side. There are human stomachs with the fundus so much developed, as to be with difficulty distinguished from those of herbivorous animals; and others, again, which approach nearer to the form of the dog's stomach from their imperfect development.

The question naturally here suggests itself:—What is the cause, not only of these differences, but of the changes in general, to which the stomach is subject at different periods of life? To me it appears that the cause is principally to be sought in the nature and quantity of the food. The cylindrical form of the stomach in children continues only while they are fed on milk, consequently on purely animal food; as soon as they receive vegetable food in any quantity, the fundus begins to develope itself. On that account, even in the first year, a strong development of the fundus is found to have taken place in such children as have been weaned immediately after their birth and fed on soft pap made of flour, potatoes, or bread. The influence of the food on the form of the stomach is distinctly observable, in older persons. The stomachs of such persons who live principally on potatoes and other vegetables are found to resemble most those of herbivorous animals; while the fundus in individuals who live more on rich animal food is less developed. I have shown in my paper, "*De Alimentorum Concoctione*, that the stomach of dogs and cats (animals purely carnivorous,) will assume the circular form after they have been fed for some time on messes of potatoes, meal, and bread; but that their stomach will retain its original oblong form if fed on animal food alone. On this account, the round form of the stomach observed in the domesticated carnivorous animals is never found in wild animals of the same class, such as, for example, the pole-cat.

Man, as an omnivorous animal, certainly possesses the type of the more rounded form of the stomach; but the extent of the development until it attain the form of the stomach of animals purely herbivorous, will, however, in a great measure, be determined by the degree of preponderance of vegetable over animal food; and the development may be increased till it become morbid. The reason why vegetable diet should develop the fundus to such a degree that the stomach assumes the circular form, (and the rotatory motion be in consequence given to it contents,) is, I believe, the following: I have shown elsewhere, in speaking of animals, that vegetable food is much more difficult of digestion, and consequently is retained much longer in the stomach. The food requires to be moved about longer, and not immediately propelled into the intestine; hence the rotary motion, by which it is agitated in the stomach without being directly emptied into the pylorus. By this action the digested part of the vegetable food is gradually separated by layers on the surface of the mass, and is conducted into the pyloric division, in order to be passed into the intestine, while the undigested part continues in rotary motion in the centre of the stomach. In carnivorous animals the process is very different: the animal food, being soon digested, is directly propelled towards the pylorus by the united action of both curvatures, and does not require to undergo a prolonged rotary motion; whereas, if vegetable food be received in a stomach so constituted, it will necessarily pass into the intestine in a raw or only partially digested state. On the other hand, herbivorous animals cannot perfectly digest animal food unless the form of the stomach undergo a change, as, by long detention in the orgun, the food, instead of being digested, becomes putrid. The attempts, therefore, which have been made in some places to feed sheep, horses, and oxen, on fish or other animal matter, must ever fail. The enquiry whether the stomach of these animals might not be transformed by gradually accustoming them to animal food, is foreign to the present subject. But even with dogs and cats, experience shows that purely vegetable food does not succeed, as it almost invariably renders them subject to the mange, (*raude.*) But to return to the cause of vomiting in children and adults.

Although the form of the stomach plays the principal part in vomiting, there seems to be another agent strongly co-operating with it, namely, the sensibility of the organ itself, particularly in respect of the nausea or sickness which produces the motions of the stomach in the act of vomiting. This is the reason why I do not assert that lunatics, who generally vomit with so much difficulty, experience this difficulty only because they have a herbivorous stomach; in such a case, we must consider the state of the brain as well as the sensibility of the stomach; the torpidity of the brain being often such as not to admit the perception of nausea; these persons, perhaps, frequently do not vomit because they do not experience nausea.

We have been endeavoring to show that the food is detained longer in the stomach of the herbivorous form, because it is kept longer in action there, without passing directly into the intestine, and that this form is adapted only to the more indigestible quality of vegetable food. If a stomach so constituted be suddenly filled with animal food, this food will be detained longer by the rotary motion than is necessary for the purpose of digestion, and the consequence will be, that the whole process will be disturbed, and the food, instead of being digested, will undergo a chemical decomposition. From this we may also conclude, that nothing will disorder the stomach sooner than sudden repletion with animal food after long use of a diet in which the vegetable preponderated. Excess of vegetable food is much less injurious in such cases, as undigested vegetable matter is, in the intestine, not so easily decomposed, and excites the peristaltic motion more than animal food. It follows that we ought carefully to avoid sudden change of diet from vegetable to animal. To this may be ascribed the greatest part of the gastric diseases prevalent in summer, and still more in autumn, when the stomach, after having been for some time accustomed to vegetable diet, is suddenly charged with large quantities of animal food.

The only remaining question, is, whether we can produce excessive retching by larger doses of emetics, as a substitute for the want of peristaltic expulsory motion in persons having stomachs of the herbivorous form? On closer observation, however, we shall be induced to believe that large doses of emetics in such cases would fail in producing the intended effect. There are persons in whom very powerful emetics would sooner produce death than vomiting, as is the case with rabbits. In such cases, I think, the greatest assistance will be afforded by such means as will facilitate vomiting, by increasing the pressure of the abdominal muscles on the stomach, such as filling it with fluids, particularly gelatinous fluids, or any thing calculated to increase the elastic tension of the parts: perhaps, after all, the best means of facilitating vomiting in stomachs of such a conformation will be starch-flower or arrow-root boiled to a paste, as formerly recommended by Hufeland.—*Hufeland and Osann's Journal, Mair,* 1835.—*British and Foreign Medical Review.*

26. *On Incontinence of Urine* By M. Mondiere.—M. Mondiere has employed the extract of nux vomica in cases of nocturnal incontinence of urine, with very beneficial effects. The case in which its efficacy was most strongly shown is that of a young woman, aged twenty, who, from the age of six years had constantly voided her urine involuntarily during the night. The use of twelve of the following pills put an end to the incontinence: they were continued until twenty-four grains of the extract had been taken, and, during the year following this treatment, there was no return of the disease. Other successful cases are mentioned.

Extracti nucis vomicæ, gr. viij.
Ferri protoxidi, gr. j. M. fiant pil. xxiv.

*Gazette Medicale. No.* 10. 1836.

*Formula for an artificial Chalybeate Water.*

℞. Ferri Sulphatis, ʒss.

Sacchar. albi, ʒiss. Misce, et divide in chart. xij. æq.

D. S. No. 1.

℞. Sodæ Carbonatis, ʒss.

Sacchar. albi, ʒiss. M. et divide in pulv. xij. æq.

D. S. No. 2.

One powder from each of these packets is to be dissolved in a small quantity of water, then mixed and drunk whilst effervescing. Each draught contains about a grain of the carbonate of the protoxide of iron, dissolved in water impregnated with carbonic acid gas, with a little Glauber's salt and carbonate of soda; the carbonate of soda being designedly a little in excess. This is a good substitute for ferruginous mineral waters, where the natural ones cannot be obtained.—*Summarium des Neuesten in der Heilkunde.* 1835.—*Ib.*

## Figures and Tables

**Fig 1. f1:**
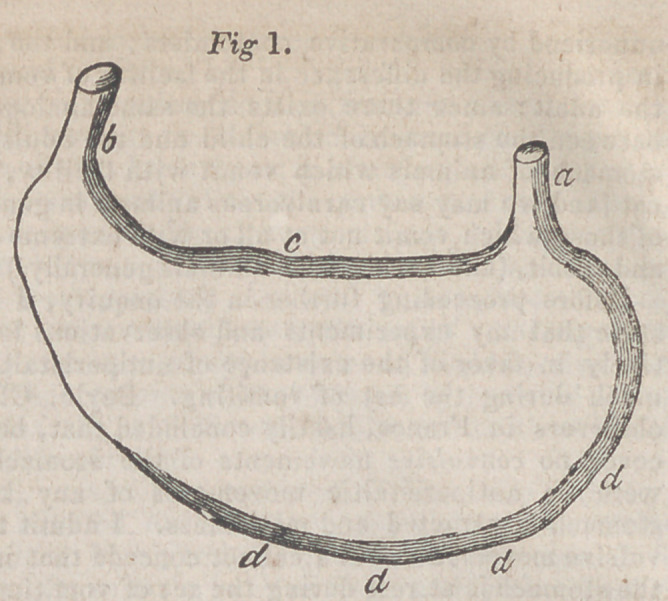


**Fig. 2. f2:**